# Direct Stereoselective Aziridination of Cyclohexenols with 3‐Amino‐2‐(trifluoromethyl)quinazolin‐4(3*H*)‐one in the Synthesis of Cyclitol Aziridine Glycosidase Inhibitors

**DOI:** 10.1002/ejoc.201801703

**Published:** 2019-01-11

**Authors:** Marta Artola, Shirley Wouters, Sybrin P. Schröder, Casper de Boer, Yurong Chen, Rita Petracca, Adrianus M. C. H. van den Nieuwendijk, Johannes M. F. G. Aerts, Gijsbert A. van der Marel, Jeroen D. C. Codée, Herman S. Overkleeft

**Affiliations:** ^1^ Department of Bio‐organic Synthesis Leiden Institute of Chemistry Leiden University Einsteinweg 55 2333 CC Leiden The Netherlands; ^2^ Department of Medical Biochemistry Leiden Institute of Chemistry Leiden University Einsteinweg 55 2333 CC Leiden The Netherlands

**Keywords:** Stereoselective aziridination, 3‐amino‐quinazolin‐4(3*H*)‐ones, Cyclophellitol, Aziridines, Glycosidases, Glycosidase inhibitors

## Abstract

Cyclophellitol aziridine and its configurational and functional isomers are powerful covalent inhibitors of retaining glycosidases, and find application in fundamental studies on glycosidases, amongst others in relation to inherited lysosomal storage disorders caused by glycosidase malfunctioning. Few direct and stereoselective aziridination methodologies are known for the synthesis of cyclophellitol aziridines. Herein, we present our studies on the scope of direct 3‐amino‐2‐(trifluoromethyl)quinazolin‐4(3*H*)‐one‐mediated aziridination on a variety of configurational and functional cyclohexenol isosters. We demonstrate that the aziridination can be directed by an allylic or homoallylic hydroxyl through H‐bonding and that steric hindrance plays a key role in the diastereoselectivity of the reaction.

## Graphical abstract

This work describes direct aziridination reactions on differently substituted glycoside configured cyclohexenes with 3‐amino‐2‐(trifluoromethyl)quinazolin‐4(3*H*)‐one as nitrogen donor. It is shown that aziridination can be directed by allylic or homoallylic hydroxyls through H‐bonding, and that steric hindrance plays an essential role in the diastereoselective outcome of the final cyclitols aziridines.

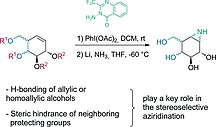

## Introduction

Glycosidases are enzymes involved in the degradation of complex glycoconjugates in nature and are of relevance both in biomedicine and biotechnology.^1^ Many glycosidases follow a two‐step Koshland double displacement mechanism, which involves a covalent enzyme‐glycoside intermediate.^2^ The active site of such retaining glycosidases is usually composed of an aspartic acid or glutamic acid, termed the catalytic acid/base, and an aspartate/glutamate (or occasionally a tyrosine) termed the nucleophile. In the first step of substrate hydrolysis, the exocyclic oxygen is protonated by the acid/base residue. Next, the catalytic nucleophile attacks at the anomeric carbon and effects an S_N_2 displacement of the aglycon, yielding a covalent enzyme–glycoside complex with inversion of the anomeric stereochemistry.

**Figure 1:**
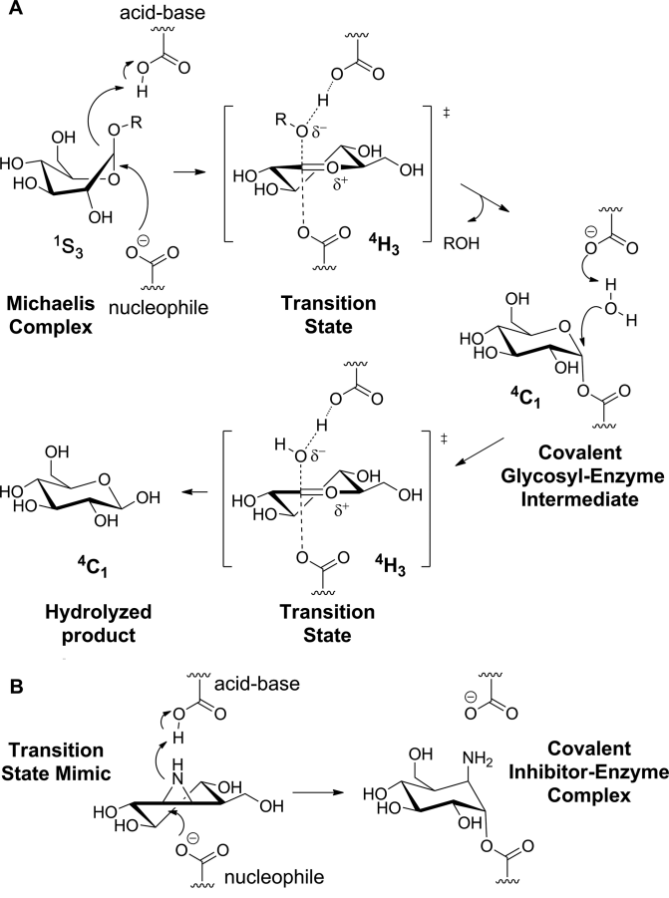
A) Koshland double displacement mechanism of retaining β‐glucosidases. B) Cyclophellitol aziridines are ^4^H_3_ transition‐state mimics and inhibit covalently and irreversibly retaining β‐glucosidases.

In the second step, a water molecule is deprotonated by the acid/base carboxylate and hydrolyses the enzyme‐substrate intermediate with a second inversion of the anomeric configuration (Figure 1A).^3^ Cyclitol aziridines can mimic the conformation of the oxocarbenium ion transition state and irreversibly inactivate glycosidases by covalently reacting with the nucleophilic carboxylate (Figure 1B). Based on this virtue and the fact, as supported by several studies in recent years from our group,^4^ that covalent and irreversible inhibition is often both very effective and highly selective, cyclitol aziridines and their corresponding activity‐based probes (ABPs) are highly useful tools for chemical glycobiology research.^5^

**Figure 2:**
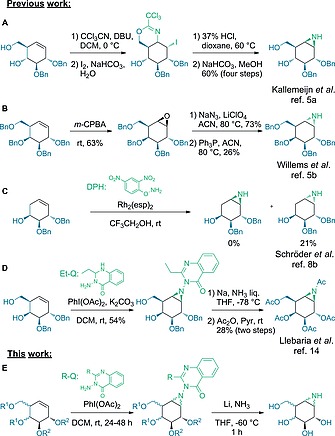
Literature aziridination reactions on glycoside configured cyclohexenes. A) Stereoselective aziridination through cyclic imidates; B) Staudinger‐like ring closure of a 1,2‐vicinal azido‐alcohol obtained after nucleophilic addition of sodium azide to an epoxide intermediate; C) Direct synthesis of unprotected N‐H aziridines with DHP and Rh_2_(esp)_2_ catalyst; D) Stereoselective aziridination by means of Et‐Q‐NH_2_ as nitrogen donor; E) Exploration of the aziridination scope by means of 3‐aminoquinazolin‐4(3*H*)‐ones (R‐Qs) on various glycoside‐configured cyclohexenols.

An important step in the synthesis of cyclitol aziridine inhibitors and ABPs involves the stereoselective aziridination of a suitable cyclohexene precursor.^6^ In contrast to epoxidations, synthetic methodologies for the direct and stereoselective aziridination of alkenes are scarce.^7^ Our previous work on the synthesis of cyclophellitol aziridines relied mostly on either intramolecular iodocyclization followed by aziridine formation (Figure 2A)[5a], 5c], 5d] or Staudinger‐type ring closure of 1,2‐azido‐alcohols obtained from an epoxide precursor (Figure 2B).[5b] We also investigated the use of *O*‐(2,4‐dinitrophenyl)hydroxylamine (DPH) as nitrogen donor with Rh_2_(esp)_2_ as catalyst (Figure 2C).^8^ However, some limitations were encountered with these methodologies. Although the iodocyclization/intramolecular substitution sequence gives complete stereochemical control with reasonable to good overall yields, this sequence is not an option when the desired aziridine has the opposite stereochemistry of that of the directing alcohol moiety. The rhodium‐catalyzed aziridination methodology appeared to be non‐stereoselective and to proceed in relatively low yields,^8^ while in the Staudinger approach, purification can be challenging and over‐reduced amino‐alcohols are occasionally generated as side products (although the use of polymer‐bound triphenylphosphine alleviates these shortcomings to certain extent[8b], 9).

In the late 1960s, Atkinson and co‐workers described the lead acetate mediated *in situ* oxidation of diverse hydrazines, amongst which 3‐aminobenzoxazolin‐2‐one was used as aziridinating agent.^10^ They initially proposed that nitrenes could be formed as intermediates^10^, ^11^ which would react with electrophilic and nucleophilic olefins to give the observed aziridines. Atkinson et al. extensively re‐examined the reactivity of different substituted 3‐aminoquinazolin‐4(3*H*)‐ones (Q‐NH_2_s) towards diverse olefins,^12^ and concluded that *N*‐acetoxy‐aminoquinazolones (Q‐NHOAc) are the reactive intermediates, rather than nitrenes.^13^ Recently, the stereospecific aziridination of a partially protected *galacto*‐configured cyclohexene has been described by Llebaria and co‐workers based on the use of 3‐amino‐2‐ethylquinazolin‐4(3*H*)‐one (Et‐Q‐NH_2_) and PhI(OAc)_2_ (PIDA). The thus formed β‐*galacto*‐configured acetylated aziridine was employed in this study to produce *N*‐aminoaziridine based irreversible inhibitors (Figure 2D).^14^ Inspired by this work of Llebaria and co‐workers we decided to explore the scope of the direct olefin aziridination reaction by investigating the reactivity of diverse aminoquinalozolin‐4‐ones towards differently configured and functionalized cyclohexenol substrates (Figure 2E). As we show here, this reaction proved to be particularly well suited for the synthesis of α‐L‐*idose*‐configured cyclophellitol aziridine, a key intermediate for the synthesis of new α‐L‐iduronidase inhibitors and ABPs, as we recently reported in a separate body of work.^15^

## Results and Discussion

**Scheme 1:**
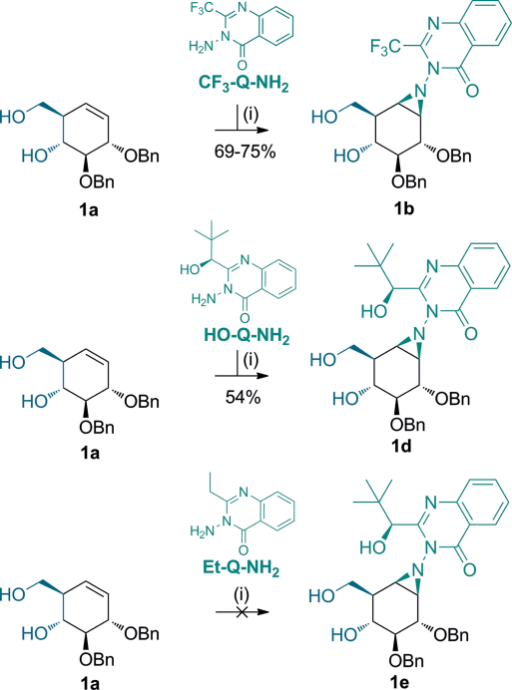
Stereoselective aziridination of cyclohexene **1a** with 3‐amino‐2‐(trifluoromethyl)quinazolin‐4(3*H*)‐one (CF_3_‐Q‐NH_2_), chiral (*S*)‐3‐amino‐2‐(1‐hydroxy‐2,2‐dimethylpropyl)quinazolin‐4(3*H*)‐one (HO‐Q‐NH_2_) or 3‐amino‐2‐ethylquinazolin‐4(3*H*)‐one (Et‐Q‐NH_2_) as nitrogen donor. Reagents and conditions: (i) PhI(OAc)_2_, R‐Q‐NH, DCM, r.t., 48 h.

**Scheme 2:**
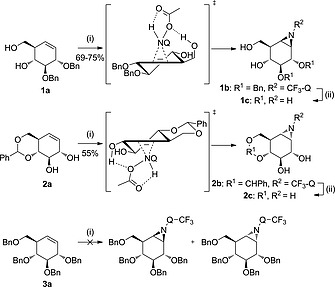
Proposed reaction transition state driven by H‐bonding from the homoallylic or allylic alcohols of gluco‐cyclohexene **1a** and **2a**, respectively, with* N*‐acetoxy‐3‐amino‐2‐(trifluoromethyl)quinazolin‐4(3*H*)‐one (CF_3_‐Q‐NHOAc, formed *in situ*) during the aziridination reactions, and subsequent deprotection under Birch conditions. Starting material was recovered when the reaction was performed on perbenzylated *gluco*‐cyclohexene **3a**. Reagents and conditions: (i) PhI(OAc)_2_, CF_3_‐Q‐NH, DCM, r.t., 48 h; (ii) Li, NH_3_ (liq.), THF, –60 °C, 1 h.

As the first research objective, d‐*gluco*‐configured cyclohexene **1a** was used as starting material to screen the most promising aminoquinazolinones described as nitrogen donors in the literature: 3‐amino‐2‐ethylquinazolin‐4(3*H*)‐one (Et‐Q‐NH_2_), 3‐amino‐2‐(trifluoromethyl)quinazolin‐4(3*H*)‐one (CF_3_‐Q‐NH_2_) and the chiral (*S*)‐3‐amino‐2‐(1‐hydroxy‐2,2‐dimethylpropyl)quinazolin‐4(3*H*)‐one (HO‐Q‐NH_2_), which all form *in situ* the reactive *N*‐acetoxy‐aminoquinazolinones in the presence of PIDA. In line with previous results,[12e] CF_3_‐Q‐NH_2_ gave superior yields (69–75 % of **1b**) when using cyclohexene **1a**, PIDA and the quinazolone in a 1:2:2 ratio respectively and by forming the reactive *N*‐acetoxy‐aminoquinazolinone at –78 °C prior to addition of the olefin at –23 °C. Aziridine intermediate **1d** was isolated in 54 % yield when using HO‐Q‐NH_2_ as aziridination agent, whereas reaction with Et‐Q‐NH_2_ returned starting material only (Scheme 1). Notably, the β‐*gluco*‐configured aziridine was formed stereoselectively in a 1.5 mmol reaction scale, indicating that hydrogen bonding from the homoallylic alcohol C7‐OH guides the incoming Q‐NHOAc, in agreement with the mechanistic proposal of Atkinson et al. (Scheme 2).[12a] These results led us to further investigate aziridinations with CF_3_‐Q‐NH_2_ on different cyclohexene substrates.

**Scheme 3:**
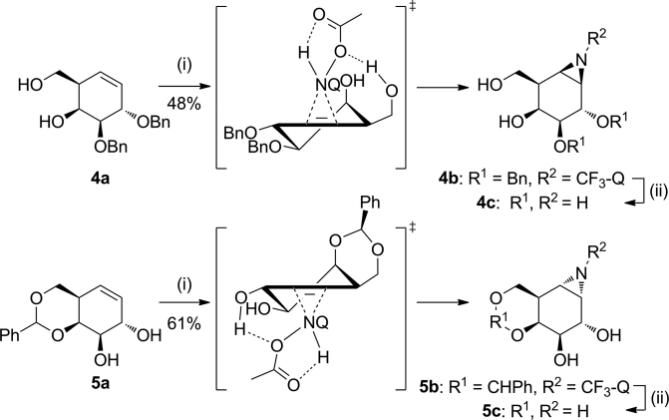
Proposed reaction transition state driven by H‐bonding from the homoallylic or allylic alcohols of *galacto‐*cyclohexene **4a** and **5a** respectively, with* N*‐acetoxy‐3‐amino‐2‐(trifluoromethyl)quinazolin‐4(3*H*)‐one (CF_3_‐Q‐NHOAc, formed *in situ*) during the aziridination reactions, and subsequent deprotection under Birch conditions. Reagents and conditions: (i) PhI(OAc)_2_, CF_3_‐Q‐NH, DCM, r.t., 48 h; (ii) Li, NH_3_ (liq.), THF, –60 °C, 1 h.

**Scheme 4:**
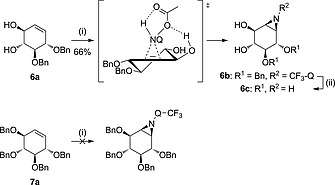
Proposed reaction transition state driven by H‐bonding from the allylic alcohol of *conduritol‐*cyclohexene **6a** with* N*‐acetoxy‐3‐amino‐2‐(trifluoromethyl)quinazolin‐4(3*H*)‐one (CF_3_‐Q‐NHOAc, formed *in situ*) during the aziridination reaction, and subsequent deprotection under Birch conditions. Perbenzylated conduritol **7a** does not react under the aziridination conditions. Reagents and conditions: (i) PhI(OAc)_2_, CF_3_‐Q‐NH, DCM, r.t., 48 h; (ii) Li, NH_3_ (liq.), THF, –60 °C, 1 h.

When *gluco*‐configured cyclohexene **2a** bearing a 4,6‐benzylidene acetal and an allylic alcohol at C‐2 was used, α‐aziridine **2b** was exclusively formed in 55 % yield, providing further support for H‐bonding guided delivery of the aziridinating reagent (Scheme 2). When perbenzylated *gluco*‐cyclohexene **3a** was subjected to the same reaction conditions no conversion was observed, indicating that the system is not reactive enough without the hydrogen bonding guided delivery and/or that the double bond, with relatively bulky substituents on either side of the alkene, is too crowded to allow for an effective addition. A similar pattern was observed with *galacto*‐configured cyclohexenes. *Galacto*‐configured cyclohexene **4a** could be stereoselectively transformed into β‐aziridine **4b** while cyclohexene **5a** afforded α‐aziridine **5b** in 61 % yield (Scheme 3). These examples again illustrate the impact of neighboring (homo)allylic alcohol functionalities. Partially protected conduritol **6a** was also amenable to stereoselective aziridination, affording β‐aziridine **6b** in 66 % yield, whereas the starting material was recovered when the reaction was performed with perbenzylated conduritol **7a** (Scheme 4).

**Scheme 5:**
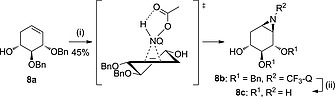
Proposed reaction transition state driven by steric hindrance of *arabino‐*cyclohexene **8a** with* N*‐acetoxy‐3‐amino‐2‐(trifluoromethyl)quinazolin‐4(3*H*)‐one (CF_3_‐Q‐NHOAc, formed *in situ*) during the aziridination reaction, and subsequent deprotection under Birch conditions. Reagents and conditions: (i) PhI(OAc)_2_, CF_3_‐Q‐NH, DCM, r.t., 48 h; (ii) Li, NH_3_ (liq.), THF, –60 °C, 1 h.

In order to investigate whether an alcohol further away from the alkene could guide the reagent to one of the diastereotopic faces of the double bond, we examined the aziridination of partially protected *xylo‐*configured cyclohexene **8a**. In this case only β‐isomer **8b** was obtained, indicating that the 4‐OH is too distal for a productive H‐bond interaction and that the aziridination takes place on the least hindered face of the double bond, opposite of the C‐2‐benzyl ether (Scheme 5).

**Scheme 6:**
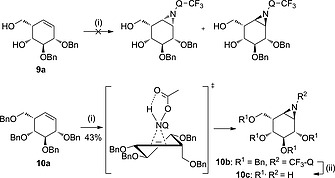
Partially protected cyclohexene** 9a** does not react under the aziridination conditions. Proposed reaction transition state driven by steric hindrance of perbenzylated *ido‐*cyclohexene **10a** with* N*‐acetoxy‐3‐amino‐2‐(trifluoromethyl)quinazolin‐4(3*H*)‐one (CF_3_‐Q‐NHOAc, formed *in situ*) during the aziridination reaction, and subsequent deprotection under Birch conditions.

We finally explored aziridination of L‐*ido‐*configured cyclohexenes in order to obtain α‐L‐*ido*‐configured aziridines as potential intermediates for the development of new iduronidase inhibitors.^15^ Partially protected cyclohexene **9a** was not amenable to aziridination, possibly because the primary alcohol directs to the beta side while this region may be hindered by the allylic benzyl ether. Considering that β‐D‐*xylo*‐configured aziridine **8a** was obtained without H‐bonding, we postulated H‐bonding would not be essential for a satisfactory aziridination in case the double bond is readily accessible. To test this hypothesis, the free alcohols in **9a** were benzylated (benzyl bromide, sodium hydride) to generate cyclohexene **10a**. From this fully protected cyclohexenol, α‐L‐aziridine **10b** was obtained in 43 % yield together with 32 % recovered starting material after direct aziridination using CF_3_‐Q‐NHOAc as the nitrogen donor (Table 1, Scheme 6). The α‐configuration of aziridine **10c** was confirmed by comparison of the experimental ^1^H NMR coupling constants with the corresponding calculated values acquired from DFT calculations.^15^

**Table 1:** Aziridination reaction and subsequent Birch reduction on various configured cyclohexenes** 1a**–**10a**

Starting material	CF_3_‐Q‐mediated aziridination	Birch deprotection
**1a**	β‐D‐*gluco* **1b**: 75 %	β‐D‐*gluco* **1c**: 99 %
**2a**	α‐D‐*gluco* **2b**: 55 %	α‐D‐*gluco* **2c**: 96 %
**3a**	–a	–a
**4a**	β‐D‐*galacto* **4b**: 48 % and 15 % S.M.	β‐D‐*galacto* **4c**: 81 %
**5a**	α‐D‐*galacto* **5b**: 61 %	α‐D‐*galacto* **5c**: 85 %
**6a**	β‐D‐conduritol **6b**: 66 %	conduritol **6c**: 99 %
**7a**	–a	–a
**8a**	β‐D‐*xylo* **8b**: 45 % and 37 % S.M.^a^	β‐D‐*xylo* **8c**: 96 %
**9a**	–a	–a
**10a**	α‐L‐*ido* **10b**: 43 % and 32 % S.M.	α‐L‐*ido* **10c**: 93 %

a Starting material (S.M.) recovered only.

In all cases, one step deprotection of the aziridine and hydroxyls in the aforementioned CF_3_‐Q functionalized aziridine intermediates was achieved under Birch conditions using lithium and liquid ammonia at –78 °C. The reactions were quenched with H_2_O and impurities derived from CF_3_‐Q precipitated and were removed by filtration. The cyclitol aziridines were finally obtained in excellent yields after cation‐exchange chromatography with Amberlite H^+^ resin to eliminate the lithium hydroxide salts (Table 1, 81–99 %).

## Conclusions

We have explored direct aziridination of both, partially protected and fully protected, configurational cyclohexenol using different substituted 3‐aminoquinazolin‐4(3*H*)‐ones. From these studies we identified 3‐amino‐2‐(trifluoromethyl)quinazolin‐4(3*H*)‐one (CF_3_‐Q) as the superior aziridinating agent. Using this reagent, direct aziridination reaction can be applied on diverse glycoside configured cyclohexenes, and it appears that aziridination can be directed by allylic or homoallylic hydroxyls through H‐bonding and that steric hindrance plays an essential role in the diastereoselectivity of the reaction. With this in mind, one could tune the cyclohexene scaffold depending on the desired configuration of the target aziridine and thus, synthesize diverse glycosidase inhibitors effectively in asymmetric fashion.

## Experimental Section

**General methods and materials:** All reagents were of a commercial grade and were used as received unless stated otherwise. Dichloromethane (DCM), tetrahydrofuran (THF) and *N*,*N*‐dimethylformamide (DMF) were stored over 4 Å molecular sieves, which were dried *in vacuo* before use. All reactions were performed under an argon atmosphere unless stated otherwise. Reactions were monitored by analytical thin‐layer chromatography (TLC) using Merck aluminum sheets pre‐coated with silica gel 60 with detection by UV absorption (254 nm) and by spraying with a solution of (NH_4_)_6_Mo_7_O_24_ **·**H_2_O (25 g/L) and (NH_4_)_4_Ce(SO_4_)_4_ **·**H_2_O (10 g/L) in 10 % sulfuric acid followed by charring at ca. 150 °C or by spraying with an aqueous solution of KMnO_4_ (7 %) and K_2_CO_3_ (2 %) followed by charring at ca. 150 °C. Column chromatography was performed manually or with a Biotage Isolera™ flash purification system using silica gel cartridges (Screening devices SiliaSep HP, particle size 15–40 µm, 60A) in the indicated solvents. ^1^H NMR and ^13^C NMR spectra were recorded on Bruker AV‐400 (400/101 MHz) and Bruker AV‐500 (500/126 MHz) spectrometer in the given solvent. Chemical shifts are given in ppm relative to the chloroform residual solvent peak or tetramethylsilane (TMS) as internal standard. Coupling constants are given in Hz. All given ^13^C spectra are proton decoupled. The following abbreviations are used to describe peak patterns when appropriate: s (singlet), d (doublet), t (triplet), q (quadruplet), m (multiplet), br (broad), Ar (aromatic), C_q_, (quaternary carbon), Q (quinazolinone). 2D NMR experiments (HSQC, COSY and NOESY) were carried out to assign protons and carbons of the new structures. High‐resolution mass spectra (HRMS) of intermediates were recorded with a LTQ Orbitrap (Thermo Finnigan) and final compounds were recorded with an apex‐QE instrument (Bruker). Optical rotations were measured on an Anton Paar MCP automatic polarimeter (Sodium D‐line, *λ* = 589 nm). LC/MS analysis was performed on an LCQ Advantage Max (Thermo Finnigan) ion‐trap spectrometer (ESI^+^) coupled to a Surveyor HPLC system (Thermo Finnigan) equipped with a C18 column (Gemini, 4.6 mm × 50 mm, 3 µm particle size, Phenomenex) equipped with buffers A: H_2_O, B: acetonitrile (MeCN) and C: 1 % aqueous TFA, or an Agilent Technologies 1260 Infinity LCMS with a 6120 Quadrupole MS system equipped with buffers A: H_2_O, B: acetonitrile (MeCN) and C: 100 mM NH_4_OAc.

2,3‐Bis‐*O*‐benzyl‐D‐*gluco*‐cyclohexene (**1a**),^1^ 7,4‐*O*‐benzylidene‐D‐*gluco*‐cyclohexene (**2a**),^16^ perbenzylated D‐*gluco*‐cyclohexene (**3a**),[4b] 2,3‐Bis‐*O*‐benzyl‐D‐*galacto*‐cyclohexene (**4a**),^17^ 2,3‐Bis‐*O*‐benzyl‐conduritol cyclohexene (**6a**),^18^ perbenzylated conduritol cyclohexene (**7a**),^16^ 2,3‐Bis‐*O*‐benzyl‐D‐*xylo*‐cyclohexene (**8a**),[8b] 2,3‐Bis‐*O*‐benzyl‐L‐*ido‐*cyclohexene (**9a**)^15^ and perbenzylated L‐*ido‐*cyclohexene (**10a**),^15^ 3‐amino‐2‐ethylquinazolin‐4(3*H*)‐one,^19^ (*S*)‐3‐amino‐2‐(1‐hydroxy‐2,2‐dimethylpropyl)quinazolin‐4(3*H*)‐one^20^ and 3‐amino‐2‐(trifluoromethyl)‐2,3‐dihydroquinazolin‐4(1*H*)‐one^19^ were synthesized following procedures previously described and their spectroscopic data are in agreement with those previously reported.

***Note*:** Numbering of proton peaks in cyclohexene and cyclitol aziridine derivatives is according to the numbering in Scheme 1.

**(3a*S*,4*S*,5*R*,7a*S*)‐5‐(hydroxymethyl)‐2‐phenyl‐3a,4,5,7a‐tetrahydrobenzo[*d*][1,3]dioxol‐4‐ol (5a):** (1*S*,2*R*,3*S*,6*R*)‐6‐(hydroxymethyl)cyclohex‐4‐ene‐1,2,3‐triol^9^, ^21^ was dissolved in dry DMF (2.0 mL) and dry MeCN (6.0 mL) in an inert atmosphere. CSA (173 mg, 0.74 mmol, 0.2 equiv.) was added to the solution, followed by PhCH(OMe)_2_ (838 µL, 5.58 mmol, 1.5 equiv.). The reaction was stirred overnight and then it was quenched with Et_3_N (100 µL, 0.23 mmol, 0.2 equiv.). The reaction mixture was extracted with EtOAc (x2) and water. The combined organic layers were washed with brine, dried with Na_2_SO_4_, filtered and concentrated in vacuo. The product was purified by silica column chromatography (from 30 %→70 % EtOAc in pentane), affording cyclohexene **5a** (598 mg, 2.41 mmol, 61 %). ^1^H NMR (500 MHz, CDCl_3_): *δ =* 7.45–7.37 (m, 2H, 2 × CH Ar), 7.33 (dd, *J* = 5.1, 2.0 Hz, 3H, 3 × CH Ar), 5.79 (dt, *J* = 10.1, 2.5 Hz, 1H, C*H*=CH), 5.55 (dd, *J* = 10.1, 2.1 Hz, 1H, C*H*=CH), 5.43 (s, 1H, C*H*‐Ph), 4.41 (d, *J* = 7.2 Hz, 1H, CH‐2), 4.29 (d, *J* = 1.4 Hz, 1H, CH‐4), 4.16 (d, *J* = 11.6 Hz, 1H, CH‐7a), 4.02 (dd, *J* = 11.7, 3.4 Hz, 1H, CH*‐*7b), 3.59 (t, *J* = 7.3 Hz, 1H, CH‐3), 3.33–3.14 (m, 2H, 2 × OH), 2.22 (s, 1H, CH‐5). ^13^C NMR (126 MHz, CDCl_3_): *δ* 138.3 (C_q_ Ph), 130.8 (C‐6), 129.2 (CH Ar), 128.4 (2 × CH Ar), 127.8 (C‐1), 126.4 (2 × CH Ar), 101.2 (CHPh), 77.0 (C‐4), 75.8 (C‐3), 70.6 (C‐2), 70.5 (C‐7), 35.8 (C‐5). ESI‐MS (*m/z*): 248.9 [M + H^+^], HRMS: calcd. for [C_14_H_17_O_4_]^+^ 249.11268, found 249.11220.

General procedure for aziridination: A solution of 3‐amino‐2‐(trifluoromethyl)‐2,3‐dihydroquinazolin‐4(1*H*)‐one (2 equiv.) in anhydrous DCM (10 mL/mmol of cyclohexene) was added dropwise over a period of 30 min to a stirred suspension of PhI(OAc)_2_ (PIDA) (2 equiv.) in anhydrous DCM (5 mL/mmol of cyclohexene) at –78 °C. The resultant mixture was stirred for additional 30 min and then cooled to –23 °C and a solution of the corresponding cyclohexene (1 equiv.) in DCM (1 mL/mmol of cyclohexene) was added dropwise over a period of 15 min. The reaction mixture was stirred at –23 °C for one hour and then the reaction was warmed to room temperature and stirred for 1–2 days. The mixture was diluted with EtOAc and subsequently washed with 0.5 m aqueous KOH solution and water. The combined water layers were extracted with EtOAc (× 2), and the combined organic layers were dried with MgSO_4_, filtered and concentrated *in vacuo*. Purification by column chromatography (from pentane to pentane/EtOAc, 1:1) gave the desired aziridines.

**β‐D‐*gluco*‐cyclitol CF_3_‐Q‐aziridine** **(1b):** Obtained from cyclohexene **1a** (487 mg, 1.40 mmol) as an orange oil in 75 % yield (609 mg, 1.07 mmol). ^1^H NMR (400 MHz, CDCl_3_): *δ =* 8.15 (dt, *J* = 8.1, 1.1 Hz, 1H, CH Q), 7.86–7.80 (m, 2H, 2 × CH Q), 7.60 (ddd, *J* = 8.2, 5.0, 3.3 Hz, 1H, CH Q), 7.40–7.30 (m, 7H, 7 × CH Ar), 7.18 (t, *J* = 7.7 Hz, 2H, 2 × CH Ar), 6.99–6.91 (m, 1H, CH Ar), 4.97 (d, *J* = 11.2 Hz, 1H, C*H*HPh), 4.75 (d, *J* = 11.4 Hz, 1H, CH*H*Ph), 4.69 (dd, *J* = 11.4, 3.4 Hz, 2H, 2 × C*H*HPh), 4.42 (dd, *J* = 7.3, 3.4 Hz, 1H, CH‐6), 4.03 (dd, *J* = 11.0, 6.7 Hz, 1H, CH‐7a), 3.92 (d, *J* = 8.2 Hz, 1H, CH‐2), 3.84 (dd, *J* = 11.0, 6.1 Hz, 1H, CH‐7b), 3.70 (d, *J* = 7.3 Hz, 1H, CH‐1), 3.50 (t, *J* = 10.0 Hz, 1H, CH‐4), 3.38 (dd, *J* = 10.0, 8.2 Hz, 1H, CH‐3), 3.16 (br s, 1H, OH), 2.96 (br s, 1H, OH), 2.14 (dtd, *J* = 10.0, 6.4, 3.4 Hz, 1H, CH‐5). ^13^C NMR (101 MHz, CDCl_3_): *δ* 161.0 (C=O), 143.9 (C_q_ Q), 143.4 (q, *J* = 35.0 Hz, *C*CF_3_), 138.2 (C_q_ Ph), 137.4 (C_q_ Ph), 135.1, 129.4, 128.6, 128.6, 128.4, 128.0, 126.5 (10 × CH Ar, 4 × CH Q), 123.2 (C_q_ Q), 118.2 (q, *J*_CF_ = 277.0 Hz, CF_3_), 84.1 (C‐3), 79.8 (C‐2), 75.1 (CH_2_Ph), 73.3 (CH_2_Ph), 69.3 (C‐4), 63.7 (C‐7), 43.6 (C‐5), 40.5, 40.4 (C‐1, C‐6). HRMS: calcd. for [C_30_H_29_F_3_N_3_O_5_]^+^ 568.20593, found 568.20551.

**β‐D‐*gluco*‐cyclitol OH‐Q‐aziridine (1d):** Obtained from cyclohexene **1a** (507 mg, 1.49 mmol) as an orange oil in 51 % yield (443 mg, 0.76 mmol). ^1^H NMR (400 MHz, CDCl_3_): *δ =* 8.21 (dd, *J* = 8.1 Hz, 1H, CH Q), 7.81–7.73 (m, 1H, CH Q), 7.71–7.66 (m, 1H, CH Q), 7.54–7.47 (m, 1H, CH Q), 7.47–7.42 (m, 2H, 2 × CH Ar), 7.37–7.30 (m, 8H, 8 × CH Ar), 5.06–5.01 (m, 2H, C*H*HPh, CH‐2), 4.99 (d, *J* = 11.1 Hz, 1H, C*H*HPh), 4.79 (s, 1H, OH), 4.76–4.69 (m, 2H, 2 × C*H*HPh), 4.27–4.06 (m, 3H, CH‐7a,b, CH‐3), 3.65–3.55 (m, 1H, OH), 3.50–3.42 (m, 2H, CH‐4, *t*Bu‐*CH*‐OH), 3.39 (dd, J = 8.1, 3.5 Hz, 1H, CH‐6), 2.76 (d, J = 8.0 Hz, 1H, CH‐1), 2.22 (ddt, J = 10.7, 7.4, 3.6 Hz, 1H, CH‐5), 1.02 (s, 9H, 3 × CH_3_).^ 13^C NMR (101 MHz, CDCl_3_): *δ* 159.4 (C=O), 144.5 (C_q_ Q), 138.1 (C_q_ Ph), 137.3 (C_q_ Ph), 134.6, 128.7, 128.6, 128.5, 128.1, 128.0, 127.9, 127.8, 127.4, 127.0, 126.6 (10 × CH Ar, 4 × CH Q), 121.2 (C_q_ Q), 84.6 (*t*Bu‐*CH*‐OH). 80.0 (C‐3), 75.4 (CH_2_Ph), 74.9 (C‐2), 72.6 (CH_2_Ph), 66.3 (C‐4), 61.3 (C‐7), 51.5 (C‐1), 49.2 (C‐6), 44.2 (C‐5), 38.5 (*C*(CH_3_)_3_), 25.9 (3 × CH_3_). ESI‐MS (*m/z*): 586.3 [M + H^+^].

**α‐D‐*gluco*‐cyclitol CF_3_‐Q‐aziridine** **(2b):** Obtained from cyclohexene **2a** (40 mg, 0.161 mmol) as an orange oil in 55 % yield (43 mg, 0.089 mmol). ^1^H NMR (500 MHz, CDCl_3_): *δ* 8.21 (d, *J* = 7.7 Hz, 1H, CH Q), 7.90–7.80 (m, 2H, 2 × CH Q), 7.62 (ddd, *J* = 8.2, 6.2, 2.1 Hz, 1H, CH Q), 7.50 (dd, *J* = 7.6, 2.1 Hz, 2H, 2 × CH Ar), 7.42–7.33 (m, 3H, 3 × CH Ar), 5.54 (s, 1H, C*H*Ph), 4.47 (dd, *J* = 10.7, 4.7 Hz, 1H, CH‐7a), 4.10 (d, *J* = 7.2 Hz, 1H, CH‐2), 4.06 (dd, *J* = 7.2, 4.5 Hz, 1H, CH‐1), 3.84 (dd, *J* = 11.8, 10.7 Hz, 1H, CH‐7b), 3.73 (dd, *J* = 10.3, 7.8 Hz, 1H, CH‐3), 3.66 (br s, 1H, OH), 3.45 (d, *J* = 7.2 Hz, 1H, CH‐6), 3.30 (t, *J* = 10.5 Hz, 1H, CH‐4), 2.85 (br s, 1H, OH), 2.38 (dddd, *J* = 12.0, 10.5, 4.7, 1.8 Hz, 1H, CH‐5). ^13^C NMR (126 MHz, CDCl_3_): *δ* 160.8 (C=O), 143.9 (C_q_ Q), 142.4 (q, *J* = 36.6 Hz, *C*CF_3_), 137.4 (C_q_ Ph), 135.4, 129.8, 129.4, 128.8, 128.5, 126.8, 126.3 (10 × CH Ar, 4 × CH Q), 122.7 (C_q_ Q), 128.2 (q, *J*_CF_ = 278.2 Hz, CF_3_), 102.3 (CHPh), 80.0 (C‐4), 73.6 (C‐3), 72.2 (C‐2), 68.8 (C‐7), 43.8 (C‐1), 41.4 (C‐6), 37.4 (C‐5). HRMS: calcd. for [C_23_H_21_F_3_N_3_O_5_]^+^ 476.1433, found 476.1423; calcd. for [C_23_H_20_F_3_N_3_NaO_5_]^+^ 498.1252, found 498.1244.

**β‐D‐*galacto*‐cyclitol CF_3_‐Q‐aziridine** **(4b):** Obtained from cyclohexene **4a** (470 mg, 2.05 mmol) as an orange oil in 49 % yield (225 mg, 1.00 mmol) and 15 % starting material recovered. [*α*]_D_^20^ = +69.4 (*c* = 0.5, CHCl_3_). ^1^H NMR (400 MHz, CDCl_3_): *δ =* 8.22–8.16 (d, *J* = 7.6 Hz, 1H, CH Q), 7.88–7.80 (m, 2H, 2 × CH Q), 7.62 (ddd, *J* = 8.2, 6.3, 2.1 Hz, 1H, CH Q), 7.42–7.21 (m, 9H, CH Ar), 7.15–7.07 (m, 1H, CH Ar), 4.93 (d, *J* = 10.9 Hz, 1H, C*H*HPh), 4.78 (d, *J* = 11.6 Hz, 1H, CH*H*Ph), 4.76 (d, *J* = 10.8 Hz, 1H, C*H*HPh), 4.67 (d, *J* = 11.8 Hz, 1H, CH*H*Ph), 4.29 (d, *J* = 8.6 Hz, 1H, CH‐2), 4.23 (dd, *J* = 11.6, 8.2 Hz, 1H, CH‐7a), 4.10 (br s, 1H, CH‐4), 3.99 (dd, *J* = 11.6, 5.8 Hz, 1H, CH‐7b), 3.94 (dd, *J* = 7.9, 3.0 Hz, 1H, CH‐6), 3.53 (br s, 1H, OH), 3.45–3.36 (m, 2H, CH‐1, CH‐3), 2.65 (d, *J* = 8.0 Hz, 1H, OH), 2.19 (br s, 1H, CH‐5). ^13^C NMR (101 MHz, CDCl_3_): *δ* 160.1 (C=O), 143.5 (C_q_ Q), 141.4 (q, *J* = 35.5 Hz, *C*CF_3_), 138.0 (C_q_ Ph), 137.9 (C_q_ Ph), 135.3, 129.8, 128.6, 128.5, 128.4, 128.3, 128.1, 127.9, 126.9 (10 × CH Ar, 4 × CH Q), 122.8 (C_q_ Q), 118.1 (q, *J*_CF_ = 275.0 Hz, CF_3_), 82.9 (C‐3), 77.5 (C‐2), 74.8 (CH_2_Ph), 71.9 (CH_2_Ph), 67.3 (C‐4), 61.9 (C‐7), 49.1 (C‐1), 44.0 (C‐6), 41.0 (C‐5). HRMS: calcd. for [C_30_H_29_F_3_N_3_O_5_]^+^: 568.2059, found 568.2061; calcd. for [C_30_H_28_F_3_N_3_NaO_5_]^+^: 590.1879, found 590.1882.

**α‐D‐*galacto*‐cyclitol CF_3_‐Q‐aziridine (5b):** Obtained from cyclohexene **5a** (550 mg, 2.2 mmol) as an orange oil in 61 % yield (643 mg, 1.35 mmol). ^1^H NMR (400 MHz, CDCl_3_): *δ* 8.18–8.09 (m, 1H, CH Q), 7.80–7.70 (m, 2H, 2 × CH Q), 7.55 (ddd, *J* = 8.3, 6.4, 2.0 Hz, 1H, CH Q), 7.40 (dq, *J* = 5.2, 2.9 Hz, 2H, 2 × CH Ar), 7.34–7.23 (m, 3H, 3 × CH Ar), 5.39 (s, 1H, CHPh), 4.37–4.23 (m, 2H, CH‐2, CH‐7a), 4.14–4.03 (m, 2H, CH‐4, CH‐7b), 3.90 (dd, *J* = 7.7, 4.1 Hz, 1H, CH‐1), 3.68–3.57 (m, 2H, CH‐3, CH‐4), 2.08 (s, 1H, CH‐5). ^13^C NMR (101 MHz, CDCl_3_): *δ* 160.3 (C=O), 143.6 (C_q_ Q), 141.8 (q, *J* = 36.0 Hz, *C*CF_3_), 137.9 (C_q_ Ph), 135.0, 129.5, 129.0, 128.4, 128.1, 126.6, 126.2 (5 × CH Ar, 5 × CH Q), 122.4 (C_q_ Q), 114.0 (q, *J*_CF_ = 277.8 Hz, CF_3_), 101.1 (CHPh), 77.5 (C‐4), 72.9 (C‐3), 69.9 (C‐7), 68.2 (C‐2), 47.7 (C‐6), 46.5 (C‐1), 34.3 (C‐5). HRMS: calcd. for [C_23_H_21_F_3_N_3_O_5_]^+^ 476.14333, found 476.14255.

**β‐D‐conduritol CF_3_‐Q‐aziridine (6b):** Obtained from cyclohexene **6a** (300 mg, 0.72 mmol) as an orange oil in 66 % yield (305 mg, 0.47 mmol). ^1^H NMR (400 MHz, CDCl_3_): *δ* 8.17 (dd, *J* = 7.8, 1.2 Hz, 1H, CH Q), 7.92–7.79 (m, 2H, 2 × CH Q), 7.61 (ddd, *J* = 8.3, 5.2, 3.2 Hz, 1H, CH Q), 7.45–7.28 (m, 12H, 12 × CH Ar), 7.28–7.14 (m, 2H, 2 × CH Ar), 7.07–6.96 (m, 1H, CH Ar), 4.97 (d, *J* = 11.3 Hz, 1H, C*H*HPh), 4.90–4.81 (m, 2H, 2 × C*H*HPh), 4.79 (d, *J* = 11.2 Hz, 1H, CH*H*Ph), 4.71 (dd, *J* = 11.3, 6.6 Hz, 2H, 2 × C*H*HPh), 4.41 (dd, *J* = 7.2, 3.7 Hz, 1H, CH‐6), 3.99 (td, *J* = 7.0, 6.1, 2.9 Hz, 2H, CH‐3, CH‐5), 3.93 (d, *J* = 7.2 Hz, 1H, CH‐1), 3.55–3.42 (m, 2H, CH‐2, CH‐4), 2.59 (br s, 1H, OH). ^13^C NMR (101 MHz, CDCl_3_): *δ* 161.0 (C=O), 143.5 (q, *J* = 34.9 Hz, *C*CF_3_), 142.9, 138.6, 138.5 (3 × C_q_ Ph), 137.6, 135.2, 129.5, 128.7, 128.6, 128.5, 128.3, 128.1, 128.0, 127.8, 126.6 (15 × CH Ar, 4 × CH Q), 123.2 (C_q_ Q), 117.3 (q, *J*_CF_ = 277.6 Hz, CF_3_), 83.6, 80.2, 79.8 (3 × CH), 75.8, 75.7, 73.9 (3 × CH_2_ Ph), 71.4 (CH), 42.6 (CH‐6), 40.6 (CH‐1). HRMS: calcd. for [C_36_H_33_F_3_N_3_O_5_]^+^: 644.2372, found 644.2368; calcd. for [C_36_H_32_F_3_N_3_NaO_5_]^+^: 666.2192, found 666.2189.

**β‐D‐*xylo‐*cyclitol CF_3_‐Q‐aziridine (8b):** Obtained from cyclohexene **8a** (300 mg, 0.97 mmol) as an orange oil in 45 % yield (233 mg, 0.43 mmol) and 37 % starting material recovered (112 mg, 0.36 mmol). ^1^H NMR (400 MHz, CDCl_3_): *δ* 8.19 (dt, *J* = 8.0, 1.1 Hz, 1H, CH Q), 7.88–7.81 (m, 2H, 2 × CH Q), 7.65–7.58 (m, 1H, CH Q), 7.43–7.29 (m, 7H, 7 × CH Ar), 7.27–7.19 (m, 2H, 2 × CH Ar), 7.07–7.00 (m, 1H, CH Ar), 4.99 (d, *J* = 11.2 Hz, 1H, C*H*HPh), 4.80 (d, *J* = 2.1 Hz, 2H, 2 × C*H*HPh), 4.67 (d, *J* = 11.2 Hz, 1H, CH*H*Ph), 4.03–3.95 (m, 2H, CH‐2, CH‐6), 3.77 (d, *J* = 7.2 Hz, 1H, CH‐1), 3.65 (td, *J* = 10.5, 5.4 Hz, 1H, CH‐4), 3.30 (dd, *J* = 10.0, 8.2 Hz, 1H, CH‐3), 2.65–2.54 (m, 2H, CH‐5a, OH), 1.74 (ddd, *J* = 14.2, 10.9, 3.5 Hz, 1H, CH‐5b). ^13^C NMR (101 MHz, CDCl_3_): *δ* 160.8 (C=O), 144.1 (C_q_ Q), 143.5 (q, *J* = 36.4 Hz, *C*CF_3_), 138.4, 137.7 (2 × C_q_ Ph), 135.0, 129.4, 128.7, 128.7, 128.5, 128.1, 128.0, 126.6 (10 × CH Ar, 4 × CH Q), 123.2 (C_q_ Q), 118.3 (q, *J*_CF_ = 275.4 Hz, CF_3_), 84.63= (C‐3), 80.4 (C‐2), 75.1 (CH_2_Ph), 73.0 (CH_2_Ph), 65.7 (C‐4), 41.4, 41.1 (C‐1, C‐6), 30.6 (C‐5). HRMS: calcd. for [C_29_H_27_F_3_N_3_O_4_]^+^: 538.1954, found 538.1960; calcd. for [C_29_H_26_F_3_N_3_NaO_4_]^+^: 560.1773, found 560.1779.

**α‐L‐*ido*‐cyclitol CF_3_‐Q‐aziridine** **(10b):** Obtained from cyclohexene **10a** (2.9 g, 5.57 mmol) as an orange oil in 43 % yield 1.79 g, 2.40 mmol). [*α*]_D_^20^ = +1.2 (*c* = 1, CHCl_3_). ^1^H NMR (400 MHz, CDCl_3_): *δ* = 8.25 (d, *J* = 7.9 Hz, 1H, CH Ar), 7.87 (d, *J* = 3.6 Hz, 2H, 2 × CH Ar), 7.67–7.63 (m, 1H, CH Ar), 7.49–7.25 (m, 19H, 19 × CH Ar), 7.14 (t, *J* = 7.4 Hz, 1H, CH Ar), 4.87 (d, *J* = 11.1 Hz, 1H, CH*H*Ph), 4.84 (s, 2H, 2 × CH*H*Ph), 4.78 (d, *J* = 11.1 Hz, 1H, CH*H*Ph), 4.74 (d, *J* = 11.6 Hz, 1H, CH*H*Ph), 4.67–4.57 (m, 3H, 3 × CH*H*Ph), 4.22 (d, *J* = 7.3 Hz, 1H, CH‐6), 4.06 (d, *J* = 7.0 Hz, 1H, CH‐3), 4.00 (d, *J* = 7.4 Hz, 1H, CH‐1), 3.98–3.96 (m, 1H, CH*H*Ph), 3.78–3.69 (m, 3H, CH‐2, CH‐4, CH*H*Ph), 2.99–2.93 (m, 1H, CH‐5).^ 13^C NMR (101 MHz, CDCl_3_): *δ* = 160.7 (C=O), 144.0 (C_q_ Ar Q), 143.4 (q, *J* = 34.1 Hz, CCF_3_), 139.0, 138.6, 138.3, 138.1 (Cq Ar), 134.9, 129.3, 128.6, 128.5, 128.4, 128.0, 127.9, 127.8, 127.7, 127.6, 126.6 (20 × CH Ar, 4 × CH Q), 123.2 (C_q_ Q), 118.2 (q,* J* = 276.68 Hz, CF_3_), 80.7 (C‐4/2), 80.2 (C‐3), 76.0 (C‐4/2), 75.2, 73.4, 73.2, 73.1 (4 × CH_2_Ph), 67.6 (C‐7), 44.2 (C‐6), 41.2 (C‐1), 38.0 (C‐5). HRMS: calcd. for [C_44_H_40_F_3_N_3_O_5_]^+^ 748.29983, found 748.29901. HRMS: calcd. for [C_44_H_40_F_3_N_3_NaO_5_]^+^ 770.28123, found 770.28076. Data in agreement with those previously reported.^15^

General procedure for aziridine deprotection: Ammonia (10 mL/mmol of starting material) was condensed at –60 °C. Lithium (15 equiv.) was added and the mixture was stirred until the lithium was completely dissolved and a bright blue solution was observed. Then, a solution of protected aziridine dissolved in anhydrous THF (5 mL/mmol) was added dropwise. The reaction mixture was stirred for 1 h at –60°C and subsequently quenched with MeOH and milliQ‐H_2_O. The solution was warmed to room temperature and stirred until the ammonia was evaporated. The reaction crude was concentrated *in vacuo*, redissolved in MilliQ‐H_2_O and filtered to remove orange solid impurities from CF_3_‐Q. The filtrate was neutralized with Amberlite IR‐120 H^+^ and the aziridine product bound to the resin was washed with water (3 times) to remove Li salts and subsequently eluted with an aqueous 1M NH_4_OH solution, and concentrated under reduced pressure to afford the fully deprotected aziridine.

**β‐D‐*gluco*‐cyclitol aziridine** **(1c):** Obtained from aziridine **1b** (479 mg, 0.84 mmol) as an oil in 99 % yield (146 mg, 0.83 mmol). ^1^H NMR (400 MHz, D_2_O):* δ* 3.94 (dd, *J* = 10.9, 4.1 Hz, 1H, CH‐7a), 3.70–3.58 (m, 2H, CH‐7b, CH‐2), 3.26 (dd, *J* = 10.2, 8.6 Hz, 1H, CH‐3), 3.02 (t, *J* = 10.1 Hz, 1H, CH‐4), 2.57 (dd, *J* = 6.1, 3.4 Hz, 1H, CH‐6), 2.29 (d, *J* = 6.2 Hz, 1H, CH‐1), 2.00 (m, 1H, CH‐5). ^13^C NMR (101 MHz, D_2_O): *δ* 76.9 (C‐3), 72.1 (C‐2), 67.7 (C‐4), 61.8 (C‐7), 43.1 (C‐5), 34.4 (C‐1), 32.5 (C‐6). Data in agreement with those previously reported.^22^

**α‐D‐*gluco*‐cyclitol aziridine (2c):** Obtained from aziridine 2b (104 mg, 0.22 mmol) as an oil in 96 % yield (37 mg, 0.21 mmol). ^1^H NMR (400 MHz, D_2_O): *δ* 3.87–3.77 (m, 2H), 3.69 (dd, *J* = 11.1, 6.4 Hz, 1H), 3.28 (dd, *J* = 10.3, 8.6 Hz, 1H), 3.19 (t, *J* = 10.1 Hz, 1H), 2.52 (dd, *J* = 6.4, 3.6 Hz, 1H), 2.30 (d, *J* = 6.4 Hz, 1H), 1.86–1.78 (m, 1H). Data in agreement with those previously reported.^23^

**β‐D‐*galacto*‐cyclitol aziridine (4c):** Obtained from aziridine 4b (100 mg, 0.18 mmol) as an orange oil in 81 % yield (25 mg, 0.14 mmol). ^1^H NMR (500 MHz, D_2_O): *δ* 3.95 (d, *J* = 8.4 Hz, 1H, CH‐2), 3.83 (dd, *J* = 7.3, 1.0 Hz, 3H, CH_2_OH, CH‐4), 3.40 (dd, *J* = 8.3, 2.4 Hz, 1H, CH‐3), 2.44–2.36 (m, 1H, CH‐1), 2.35–2.27 (m, 1H, CH‐6), 2.22–2.11 (m, 1H, CH‐5). ^13^C NMR (126 MHz, D_2_O): *δ* 76.0 (C‐3), 70.4 (C‐2), 70.1 (C‐4), 61.2 (CH_2_), 39.2 (C‐5), 34.4 (C‐6), 31.9 (C‐1). Data in agreement with those previously reported.^9^

**α‐D‐*galacto*‐cyclitol aziridine (5c):** Obtained from aziridine **5b** (622 mg, 1.31 mmol) as an oil in 85 % yield (195 mg, 1.11 mmol). ^1^H NMR (400 MHz, D_2_O): *δ* 4.05 (dd, *J* = 9.0, 4.3 Hz, 1H, CH‐2), 3.82 (dt, *J* = 3.2, 1.6 Hz, 1H, CH‐4), 3.77–3.65 (m, 2H, CH‐7a,b), 3.32 (dd, *J* = 9.0, 1.9 Hz, 1H, CH‐3), 2.57 (dd, *J* = 6.4, 4.2 Hz, 1H, CH‐1), 2.12 (dt, *J* = 6.3, 1.3 Hz, 1H, CH‐6), 1.93 (tdd, *J* = 7.5, 3.2, 1.3 Hz, 1H, CH‐5). ^13^C NMR (126 MHz, D_2_O): *δ* 72.6 (C‐3), 71.4 (C‐4), 69.0 (C‐2), 61.5 (C‐7), 42.6 (C‐5), 35.3 (C‐1), 31.4 (C‐6). Data in agreement with those previously reported.[5b]

Conduritol aziridine (6c): Obtained from aziridine 6b (100 mg, 0.16 mmol) as an oil in 99 % yield (25 mg, 0.16 mmol). 1H NMR (500 MHz, D_2_O): *δ* 3.90–3.84 (m, 1H, CH‐3), 3.71–3.67 (m, 1H, CH‐5), 3.25–3.18 (m, 2H, CH‐2, CH‐4), 2.61 (dd, *J* = 6.2, 3.6 Hz, 1H, CH‐6), 2.34 (d, *J* = 6.2 Hz, 1H, CH‐1). ^13^C NMR (126 MHz, D_2_O): *δ* 78.2 (C‐2/4), 74.7 (C‐5), 74.1 (C‐3), 73.5 (C‐2/4), 37.9 (C‐6), 37.4 (C‐1). Data in agreement with those previously reported.^24^

**β‐D‐*xylo*‐cyclitol aziridine (8c):** Obtained from aziridine **8b** (115 mg, 0.21 mmol) as an oil in 96 % yield (30 mg, 0.20 mmol). ^1^H NMR (400 MHz, D_2_O): *δ* 3.61 (d, *J* = 8.3 Hz, 1H, CH‐2), 3.34 (td, 1H,* J* = 10.5, 5.4 Hz, CH‐4), 3.15 (dd, *J* = 10.3, 8.4 Hz, 1H, CH‐3), 2.42 (br s, 1H, CH‐6), 2.31 (ddd, *J* = 13.8, 5.4, 1.6 Hz, 1H, CH‐5a), 2.17 (d, *J* = 6.1 Hz, 1H, CH‐1), 1.66 (ddd, *J* = 14.0, 10.7, 3.3 Hz, 1H, CH‐5b). ^13^C NMR (101 MHz, D_2_O): *δ* 77.5 (C‐3), 72.6 (C‐2), 66.2 (C‐4), 34.5 (C‐1), 31.3 (C‐5), 30.8 (C‐6). Data in agreement with those previously reported.[8b]

**α‐L‐*ido*‐cyclitol aziridine (10c):** Obtained from aziridine **10b** (1.79 g, 2.39 mmol) as an oil in 93 % yield (389 mg, 2.22 mmol).

^1^H NMR (400 MHz, D_2_O): *δ* 2.47 (dd, *J* = 11.2, 4.4 Hz, 1H, CH‐7a), 2.28–2.17 (m, 2H, CH‐7b, CH‐3), 2.11 (dd, *J* = 10.5, 5.6 Hz, 1H, CH‐4), 1.98 (dd, *J* = 10.5, 7.4 Hz, 1H, CH‐2), 1.07 (d, *J* = 6.0 Hz, 1H, CH‐6), 1.01 (dt, *J* = 5.8, 5.1 Hz, 1H, CH‐5), 0.77 (d, *J* = 6.0 Hz, 1H, CH‐1). ^13^C NMR (101 MHz, D_2_O): *δ =* 76.0 (C‐2), 75.2 (C‐3), 70.1 (C‐4), 62.2 (C‐7), 43.5 (C‐5), 36.9 (C‐1), 36.3 (C‐6). HRMS: calcd. for [C_7_H_14_NO_4_]^+^ 176.09228, found 176.09175. Data in agreement with those previously reported.^15^

**Conflict of interest**

The authors declare no conflict of interest.

## Supporting information

Supporting Information
