# Supplementary material for: Direct Stereoselective Aziridination of Cyclohexenols with 3‐Amino‐2‐(trifluoromethyl)quinazolin‐4(3H)‐one in the Synthesis of Cyclitol Aziridine Glycosidase Inhibitors
Source: European J Org Chem. 2019 Jan 11;2019(6):1397–404. doi: 10.1002/ejoc.201801703 (PMC6876648; doi:10.1002/ejoc.201801703)

European Journal of Organic Chemistry

Supporting Information

Direct Stereoselective Aziridination of Cyclohexenols with 3-Amino-2-(trifluoromethyl)quinazolin-4(3*H*)-one in the Synthesis of Cyclitol Aziridine Glycosidase Inhibitors

Marta Artola,* Shirley Wouters, Sybrin P. Schröder, Casper de Boer, Yurong Chen, Rita Petracca, Adrianus M. C. H. van den Nieuwendijk, Johannes M. F. G. Aerts, Gijsbert A. van der Marel, Jeroen D. C. Codée, and Herman S. Overkleeft*

ejoc201801703-sup-0001-SupMat.pdf

TABLE OF CONTENTS

^{1}H and ^{13}C NMR SPECTRA……………………………………………………………………………………………..S2-S17

NMR SPECTRA

^{1}H-NMR and ^{13}C-NMR spectra of **1b** in $CDCl_3$

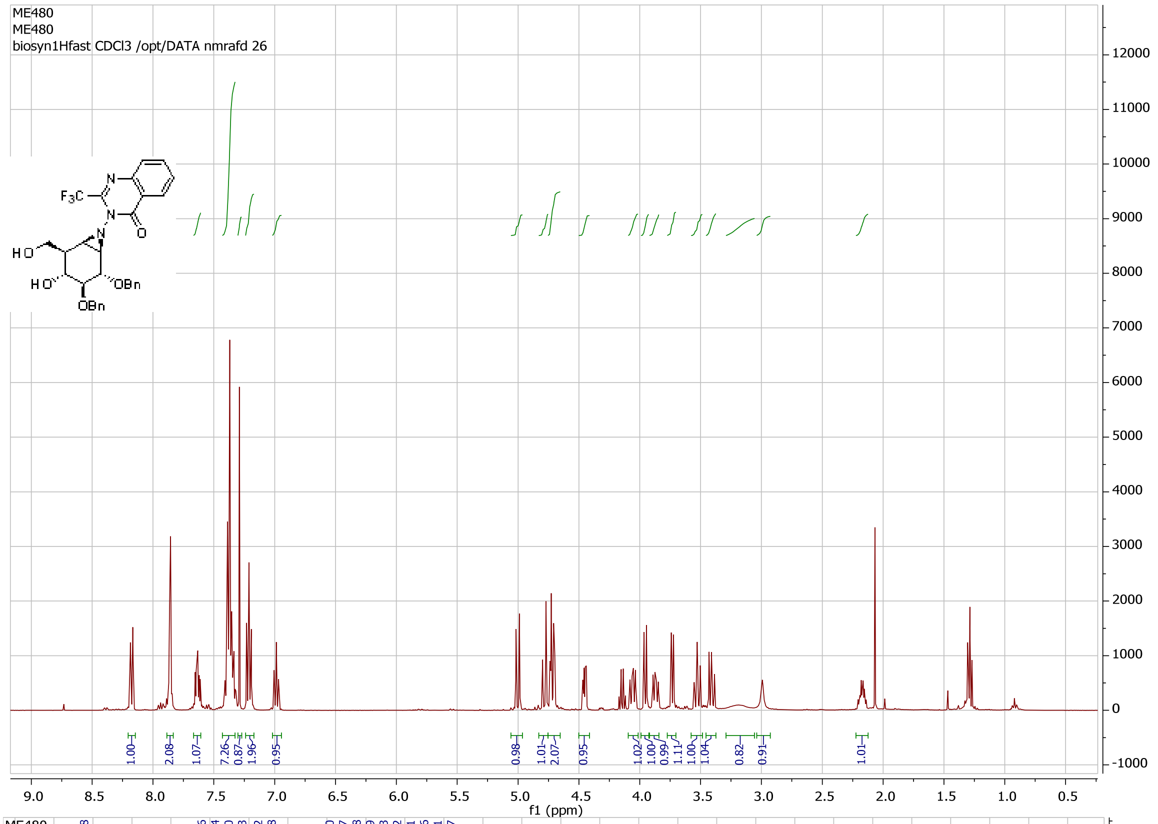

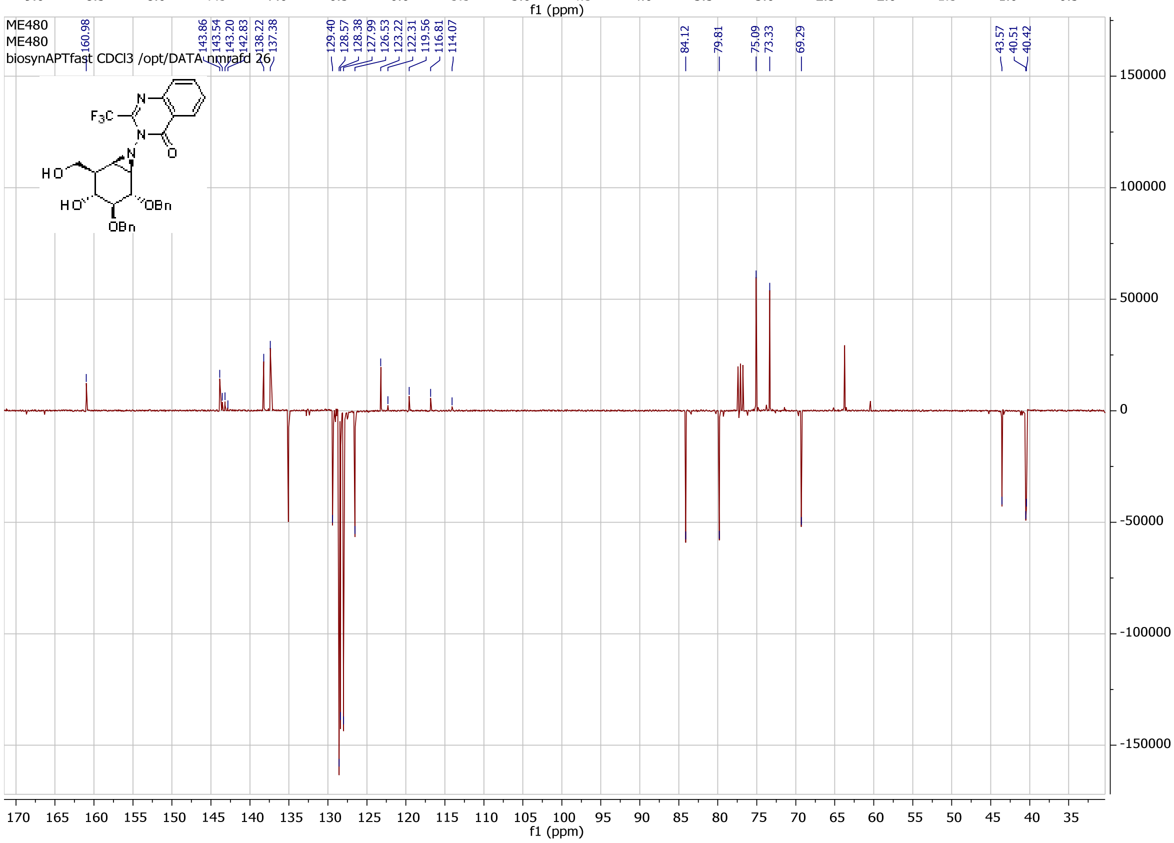

^{1}H-NMR and ^{13}C-NMR spectra of **1c** in D_2O

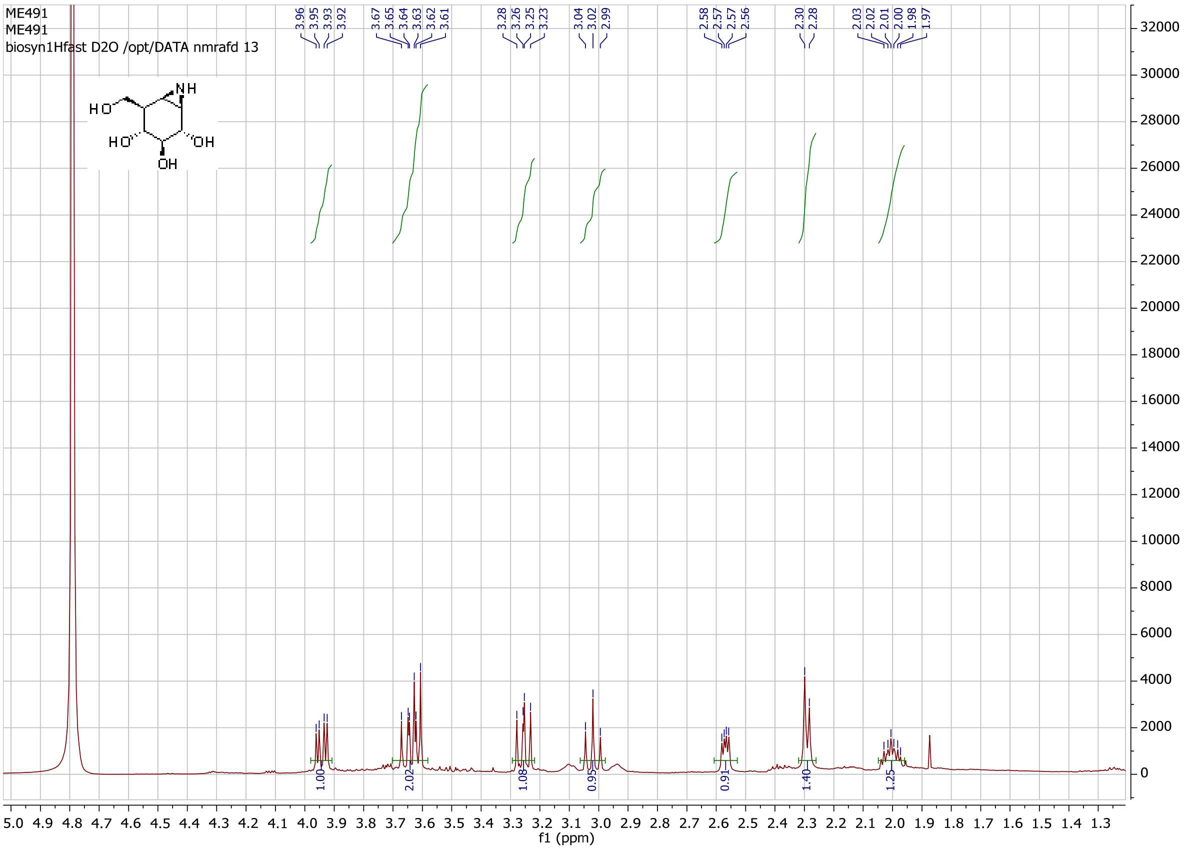

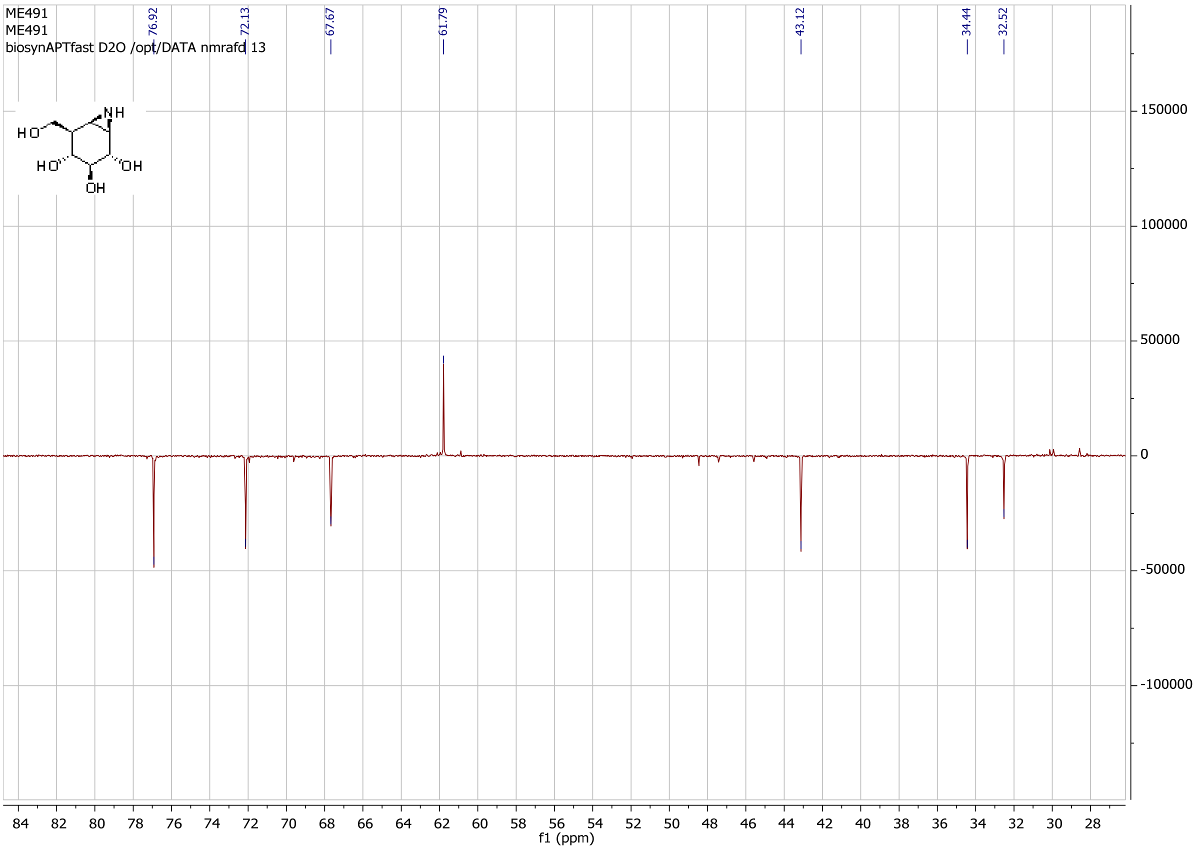

^{1}H-NMR and ^{13}C-NMR spectra of **1d** in $CDCl_3$

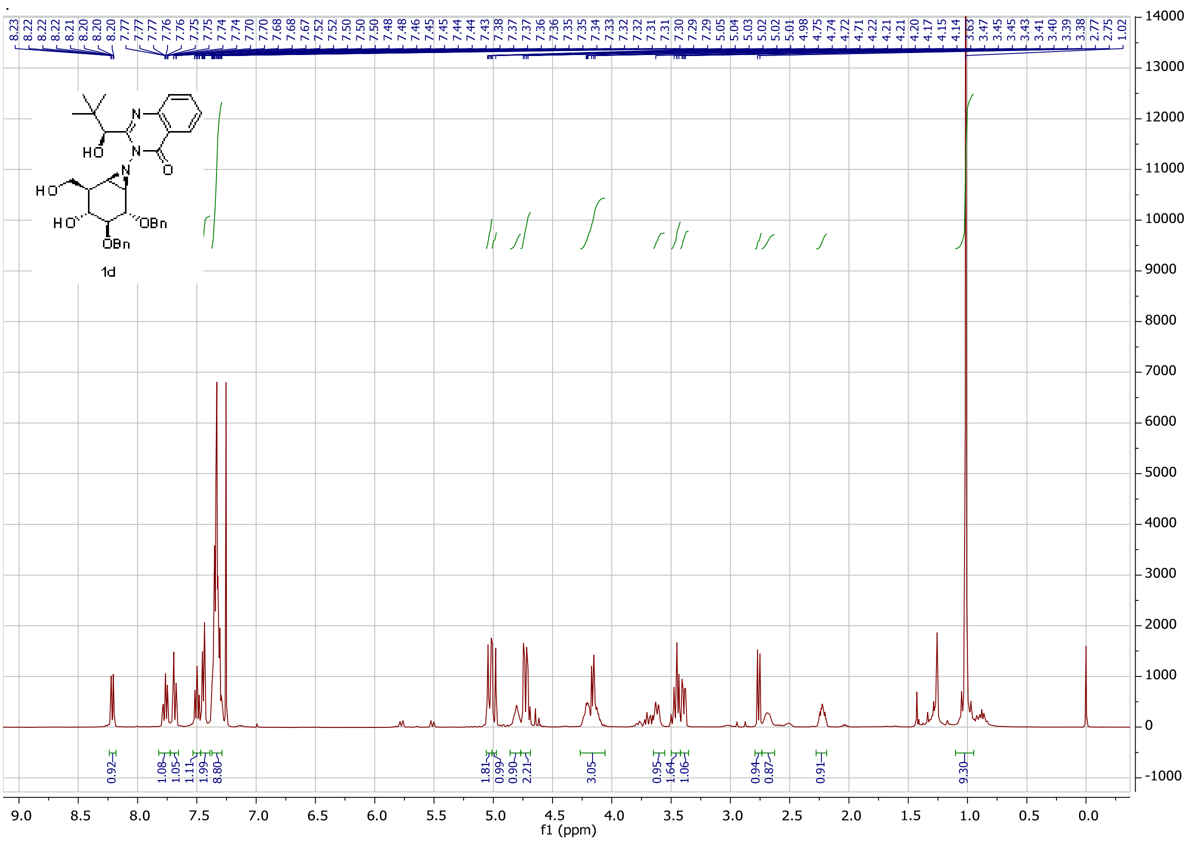

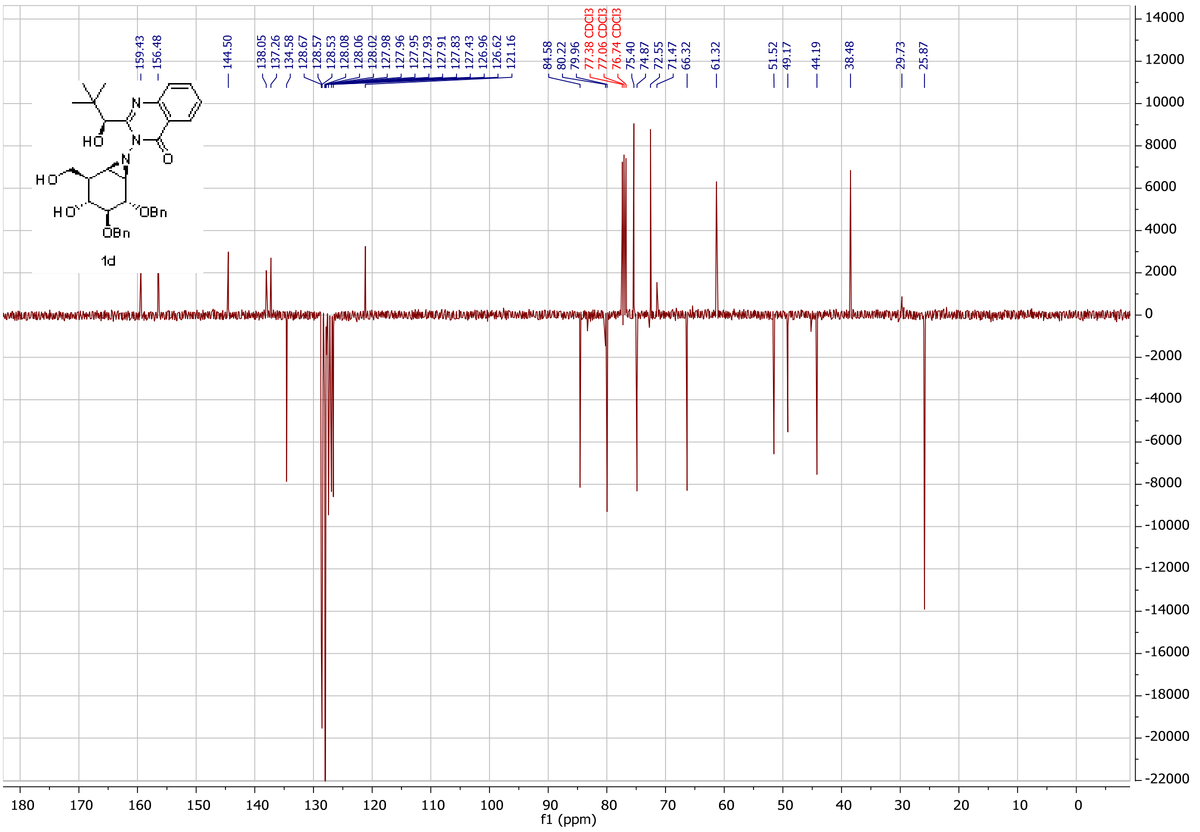

^{1}H-NMR and ^{13}C-NMR spectra of **2b** in $CDCl_3$

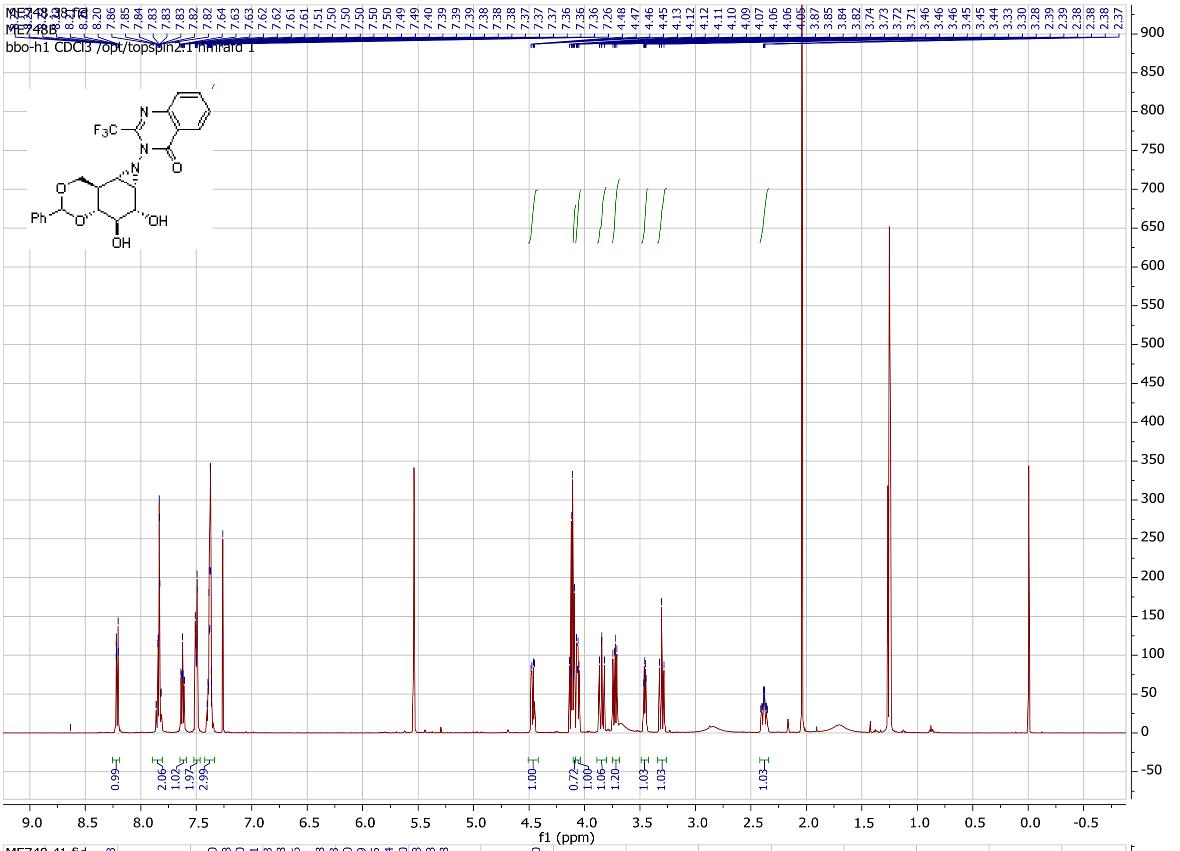

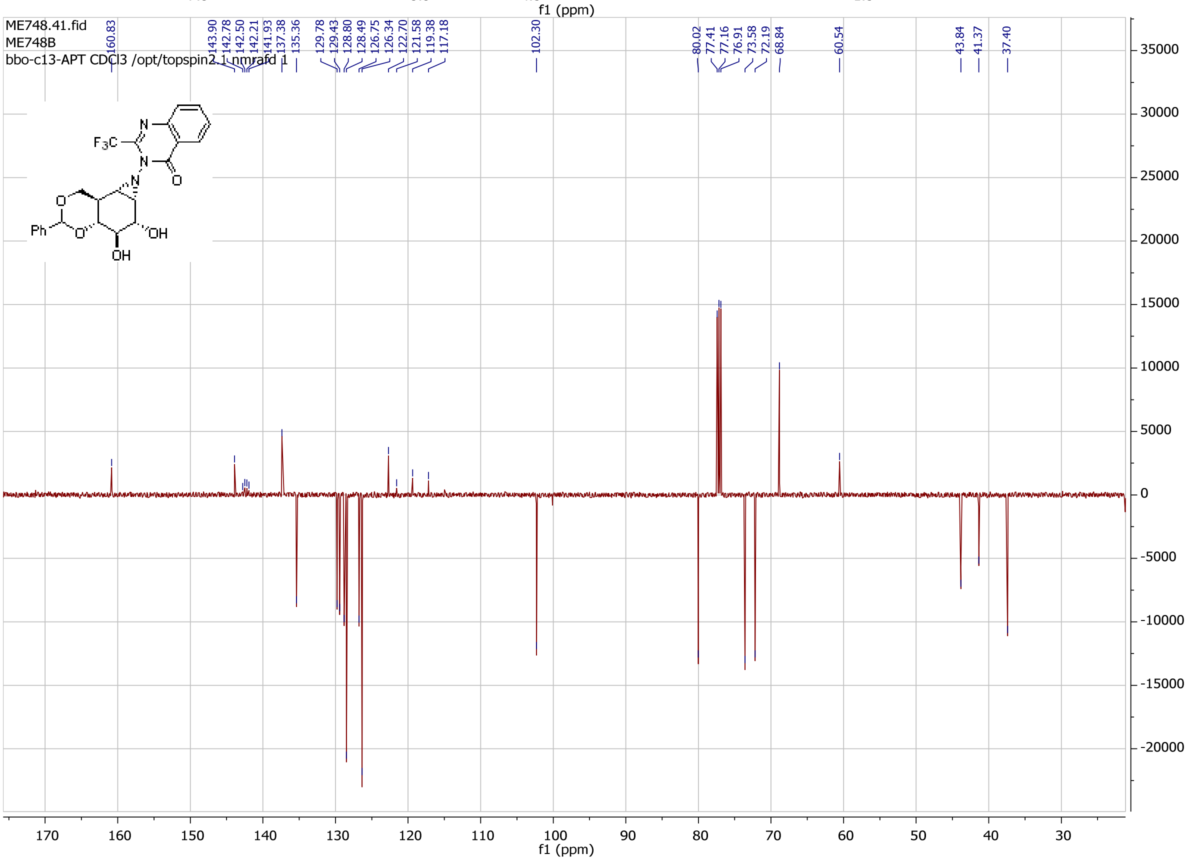

^{1}H-NMR spectra of **2c** in D_2O

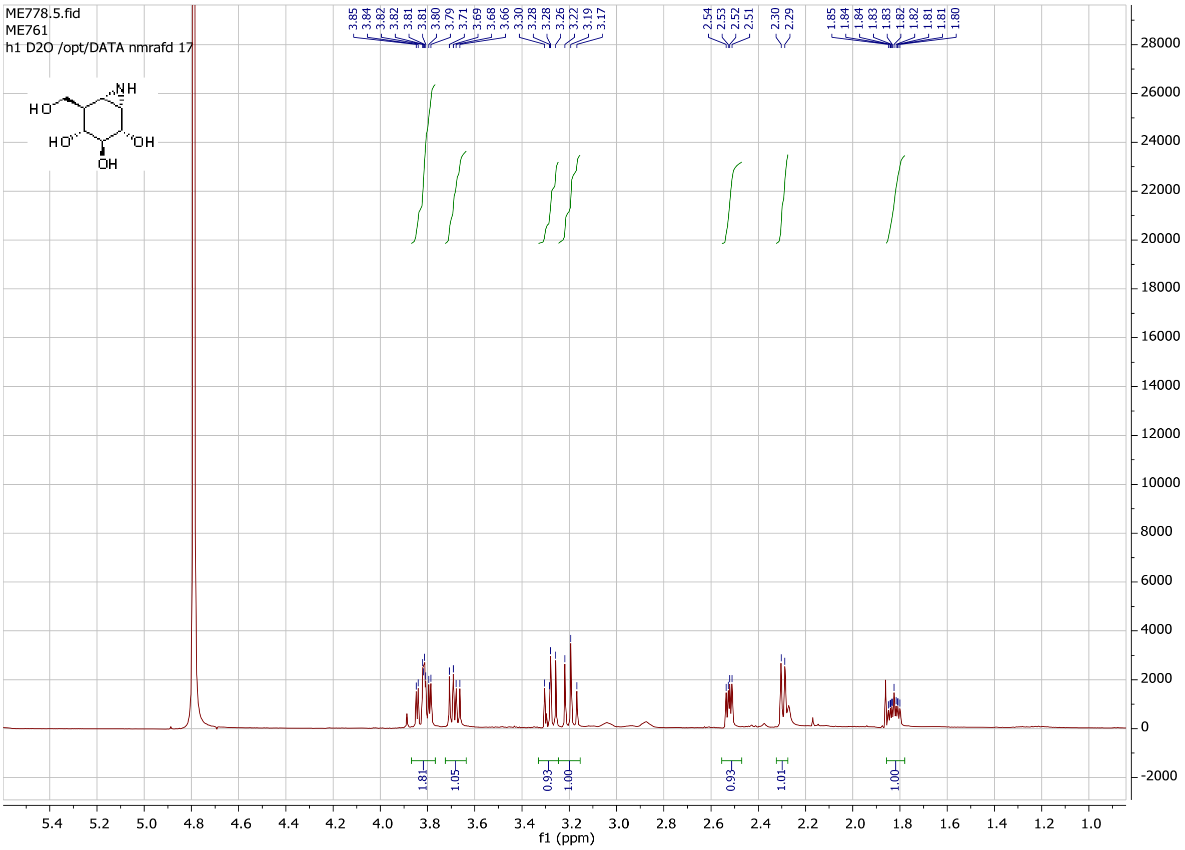

^{1}H-NMR and ^{13}C-NMR spectra of **4b** in $CDCl_3$

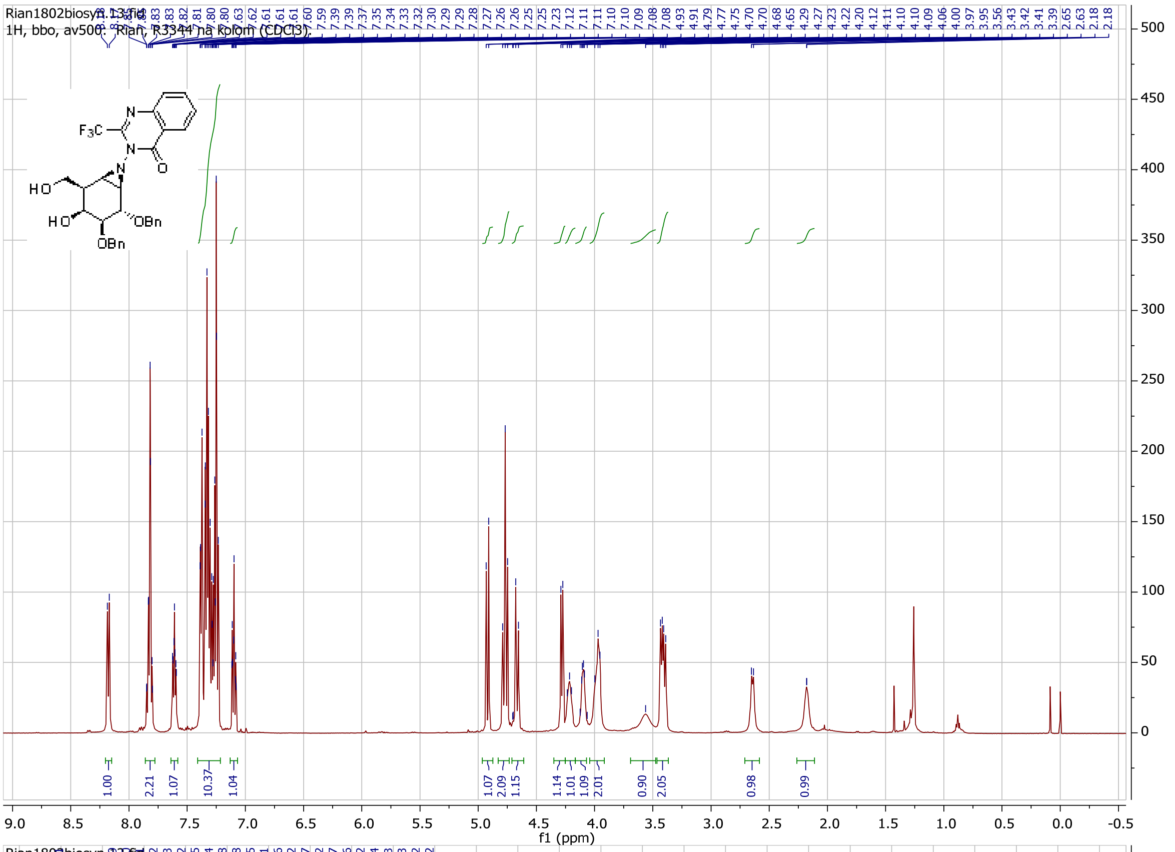

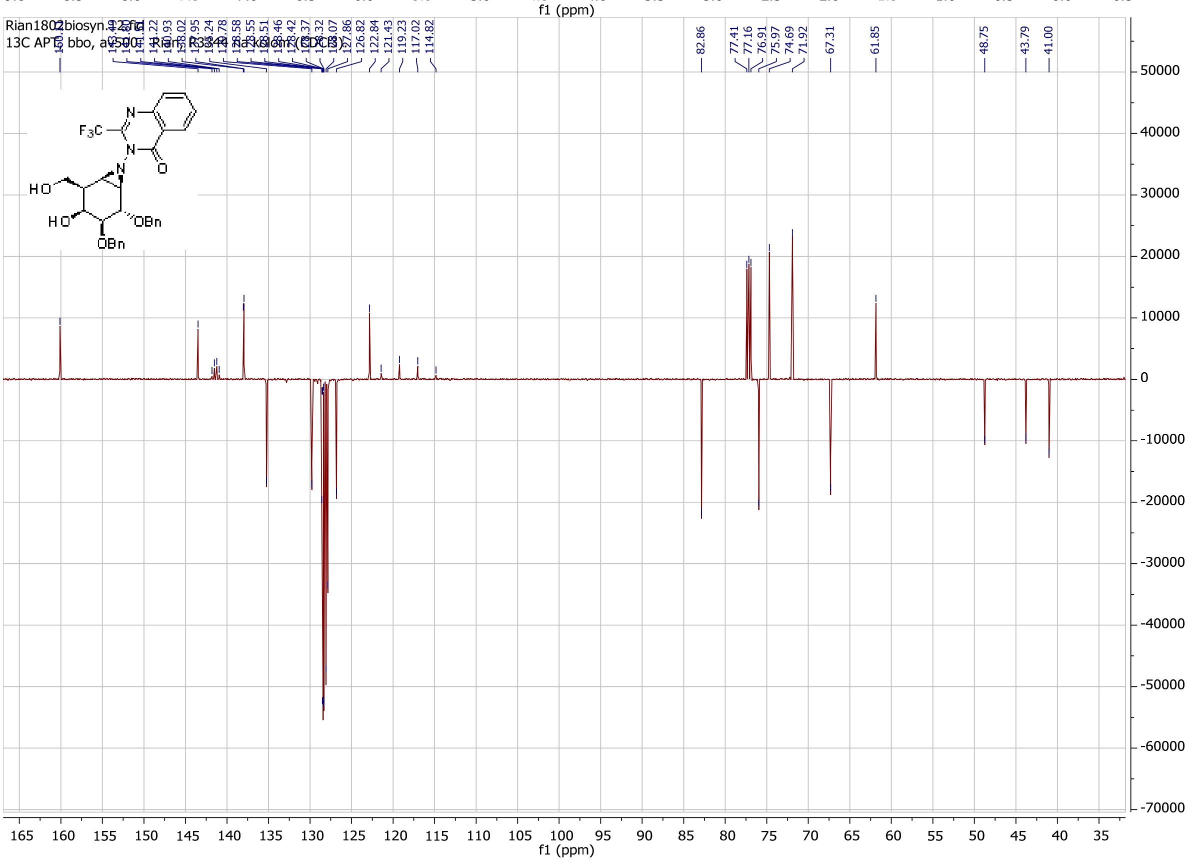

^{1}H-NMR and ^{13}C-NMR spectra of **4c** in D_2O

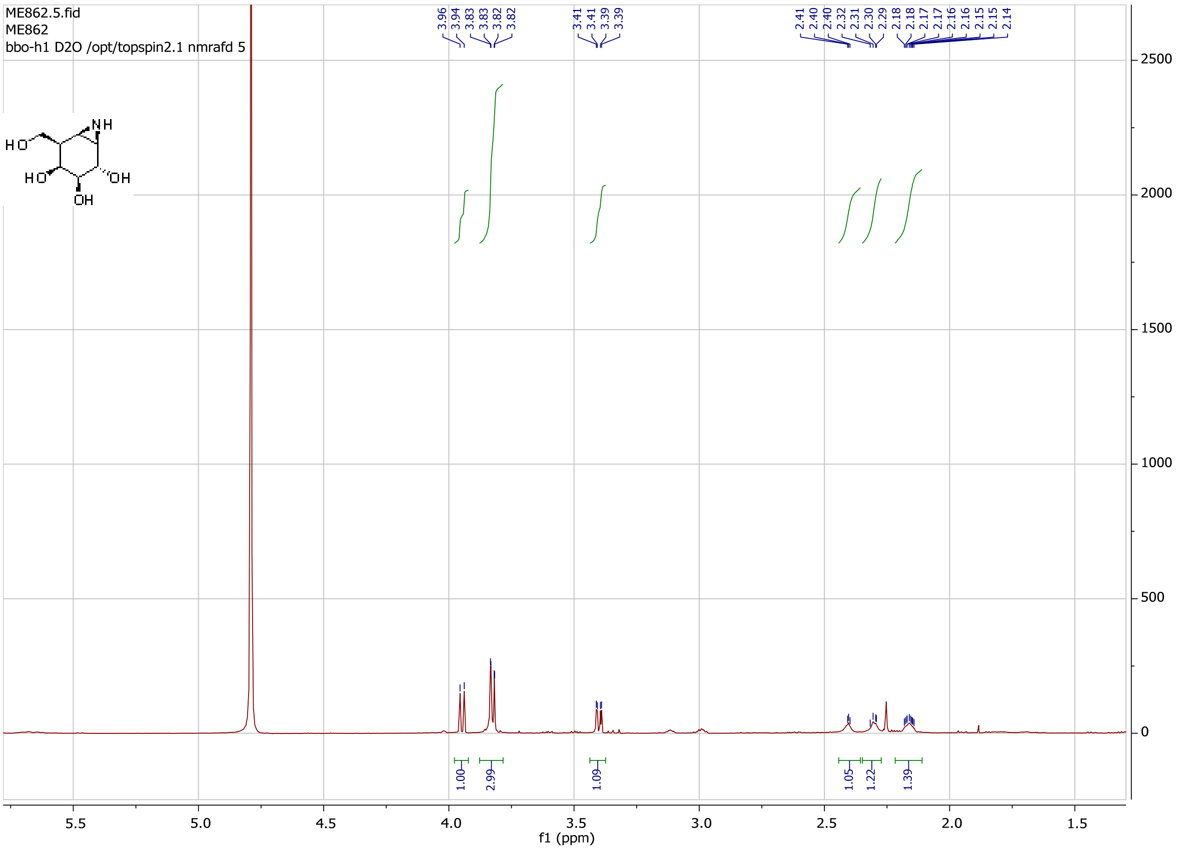

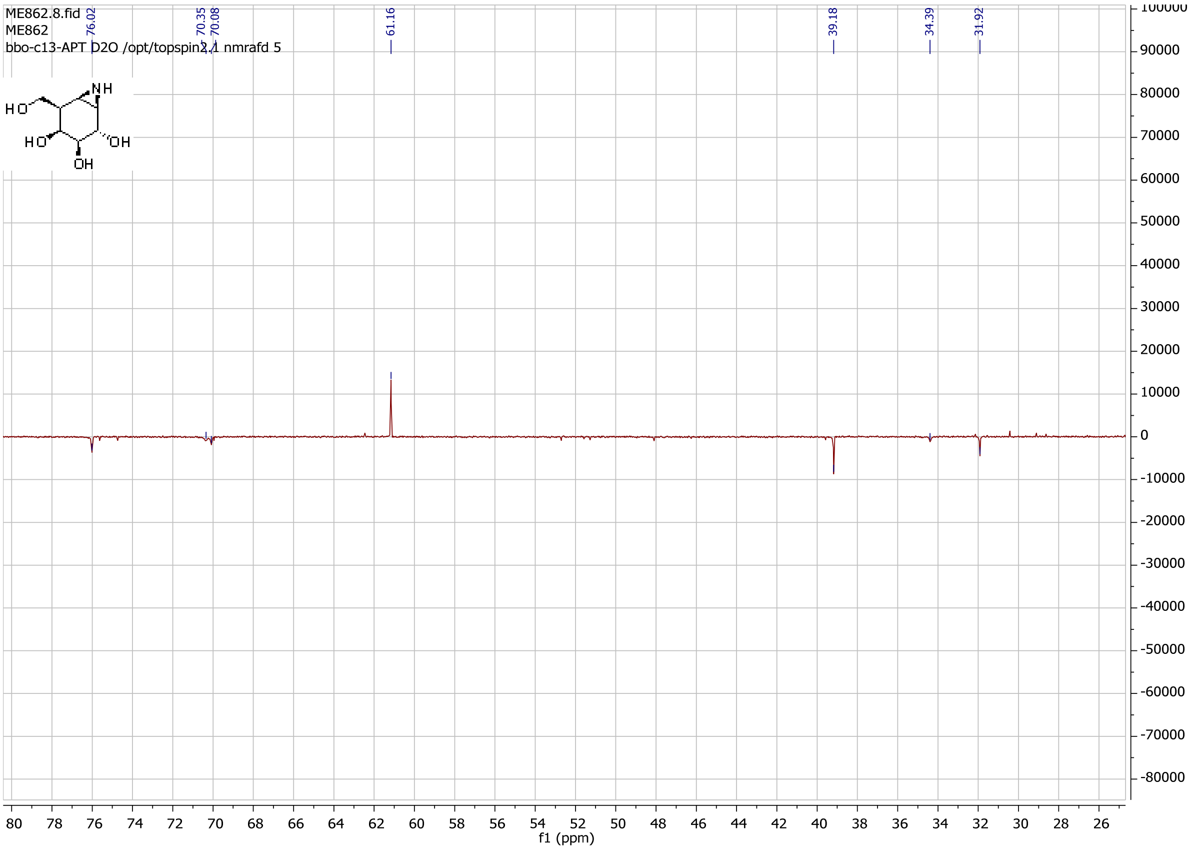

^{1}H-NMR and ^{13}C-NMR spectra of **5a** in $CDCl_3$

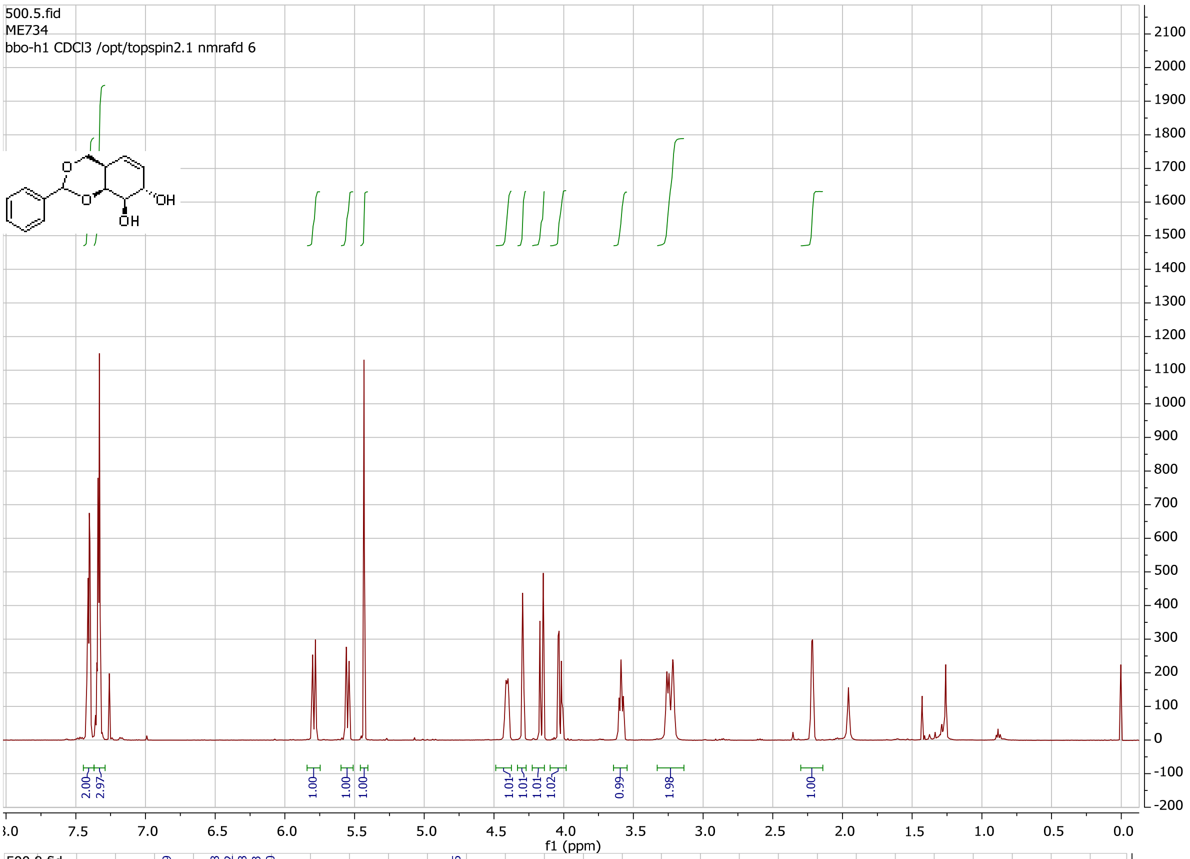

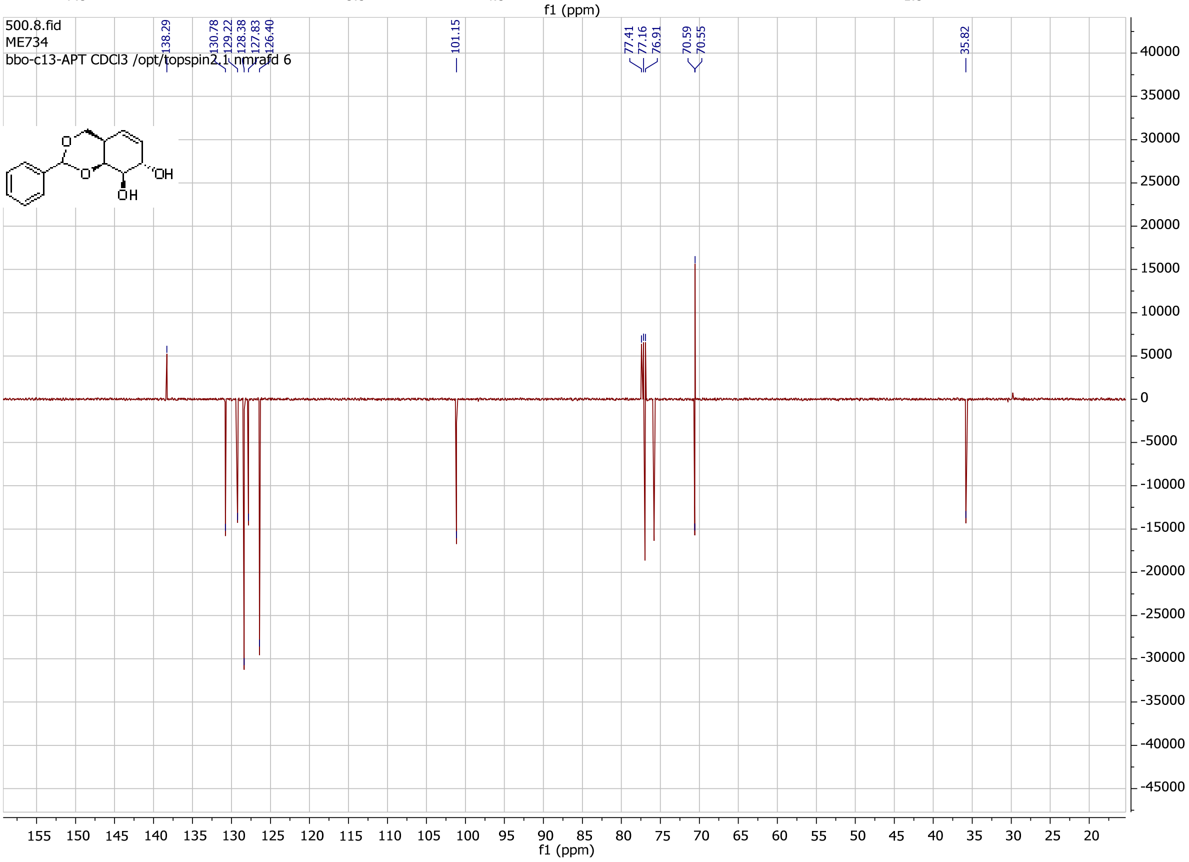

^{1}H-NMR and ^{13}C-NMR spectra of **5b** in $CDCl_3$

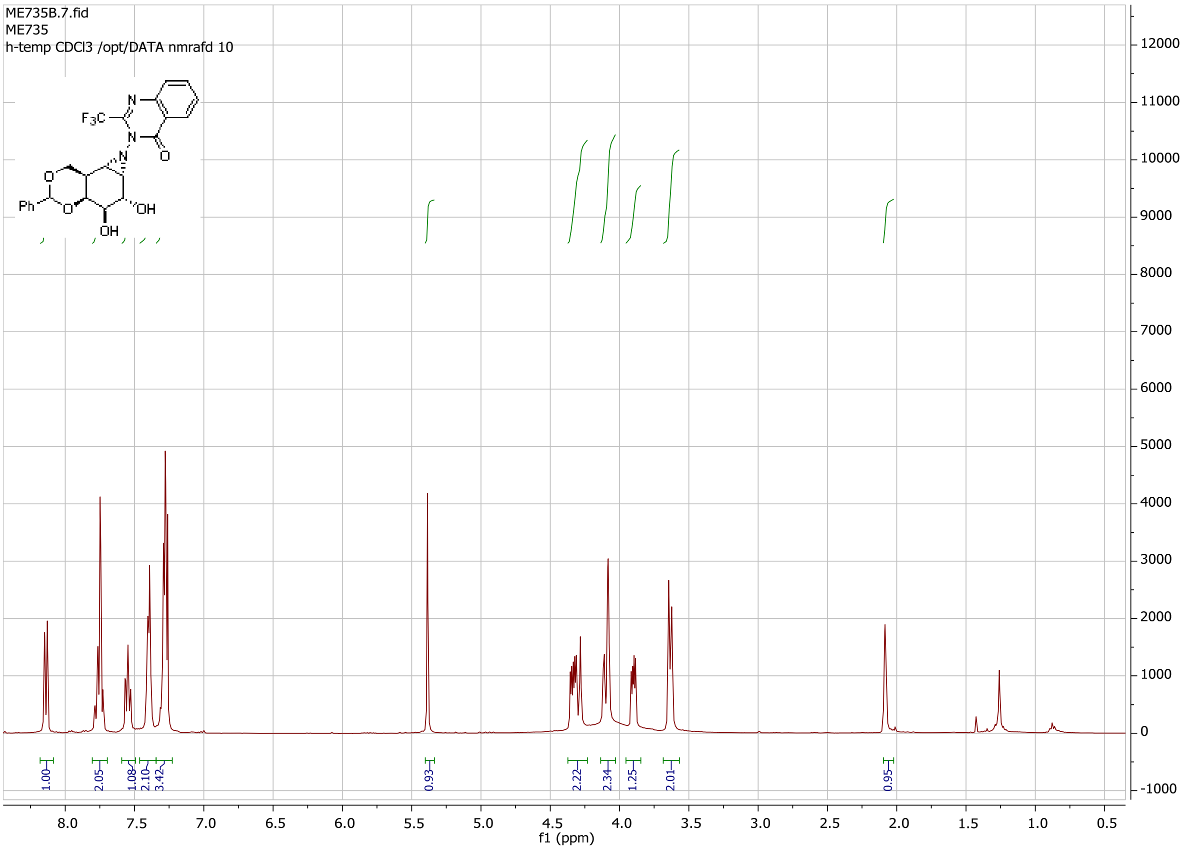

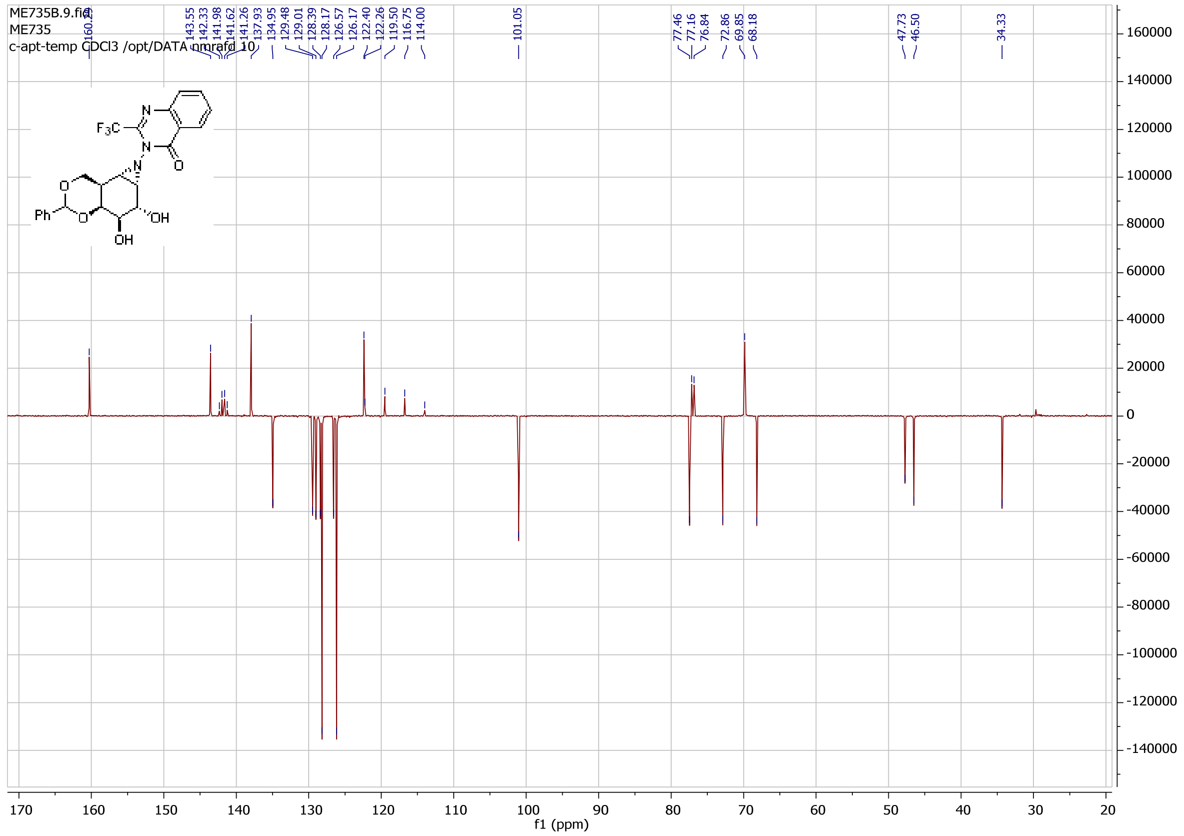

^{1}H-NMR and ^{13}C-NMR spectra of **5c** in D_2O

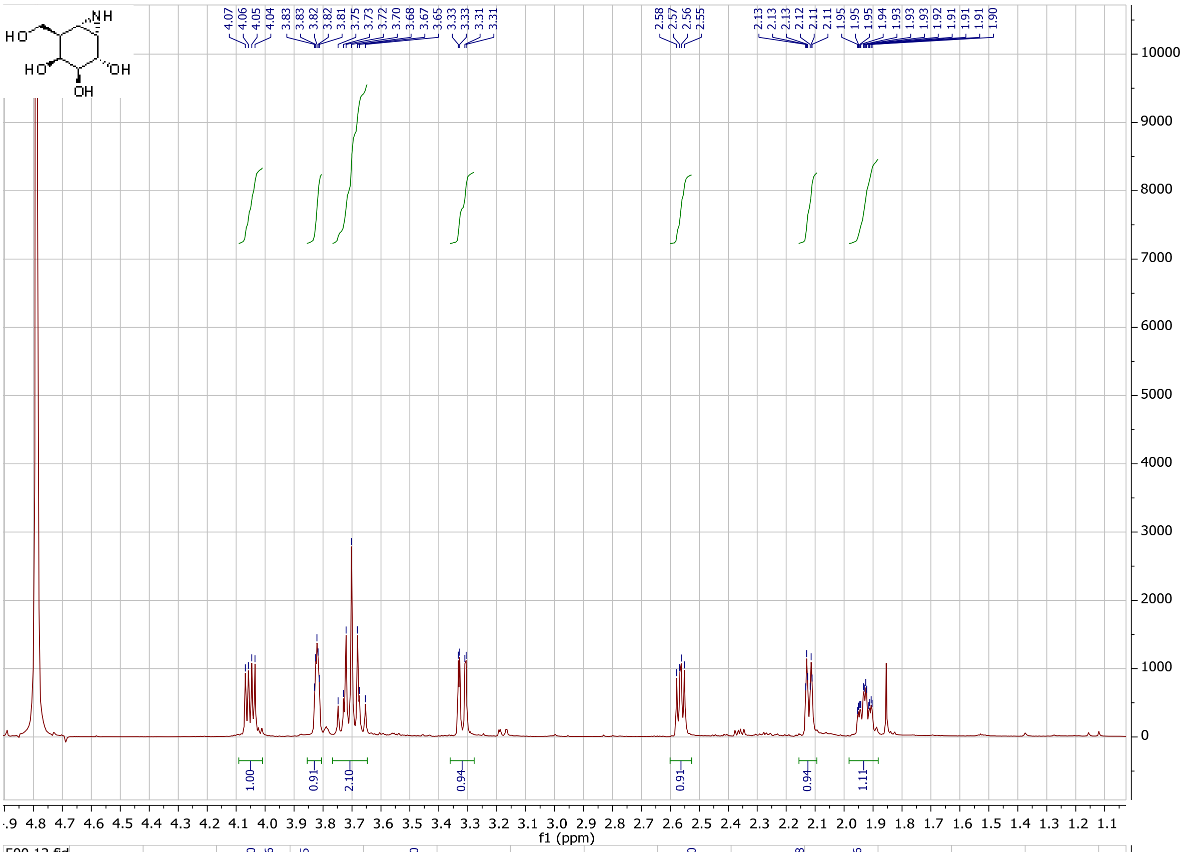

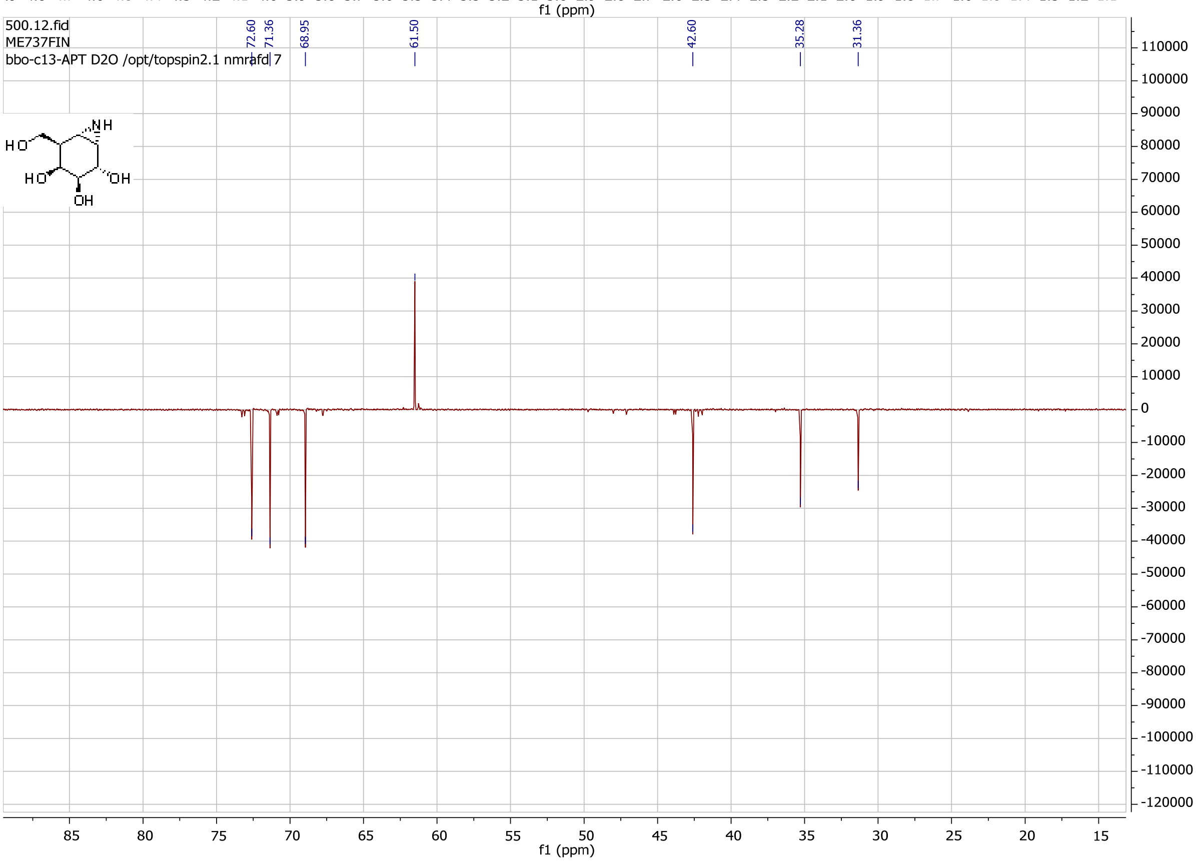

^{1}H-NMR and ^{13}C-NMR spectra of **6b** in $CDCl_3$

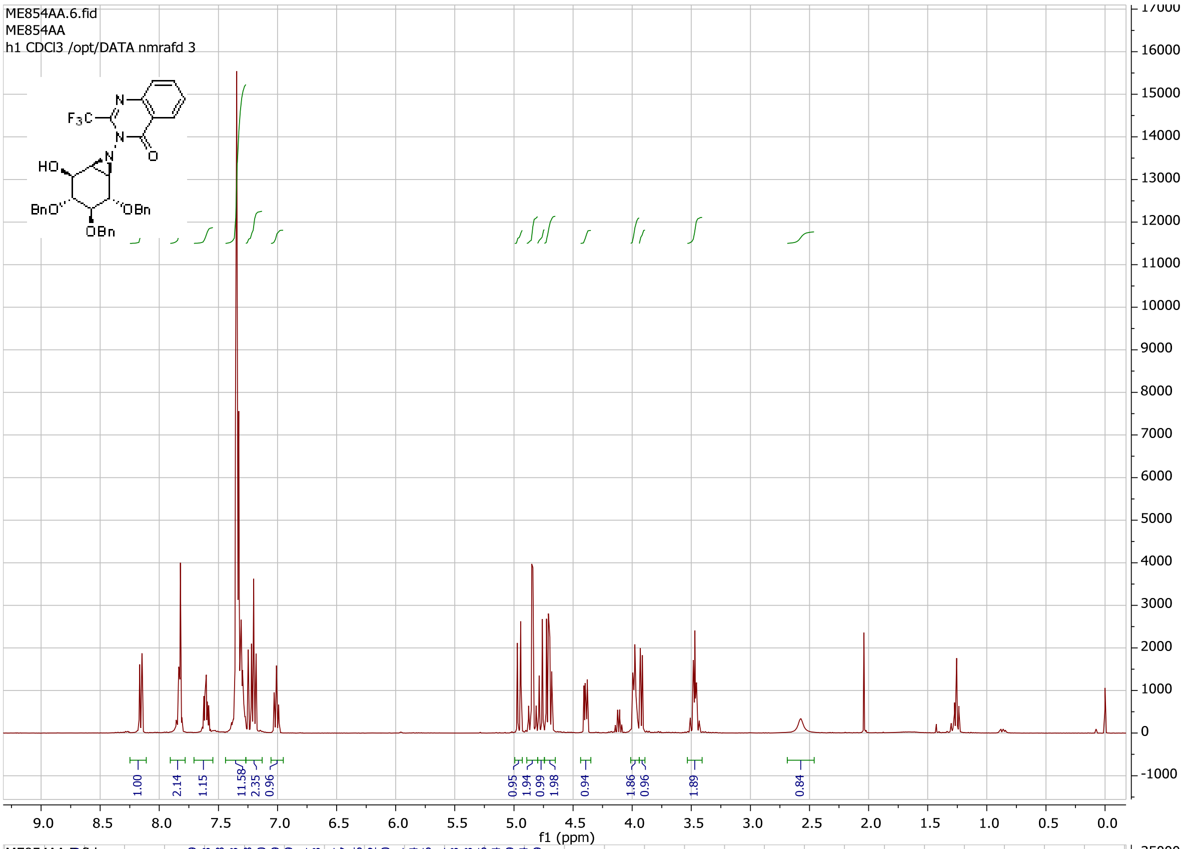

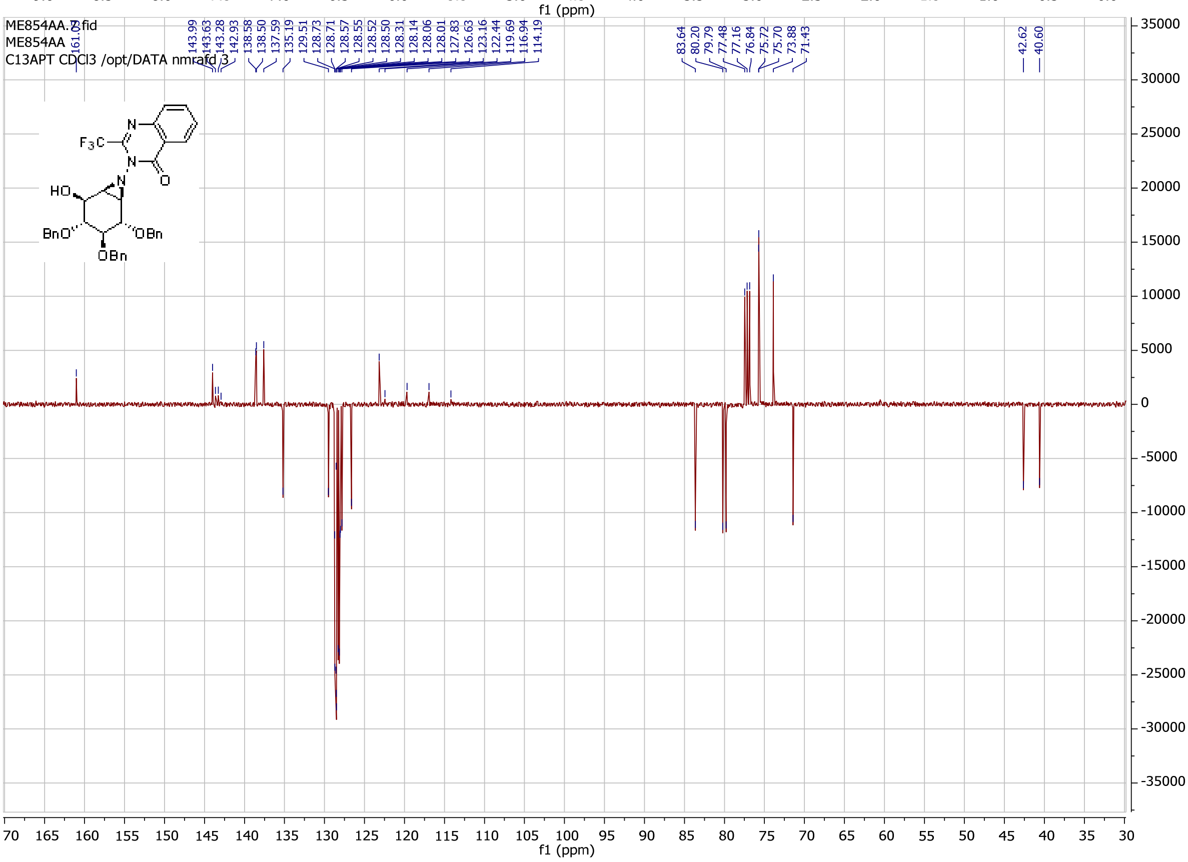

^{1}H-NMR and ^{13}C-NMR spectra of **6c** in D_2O

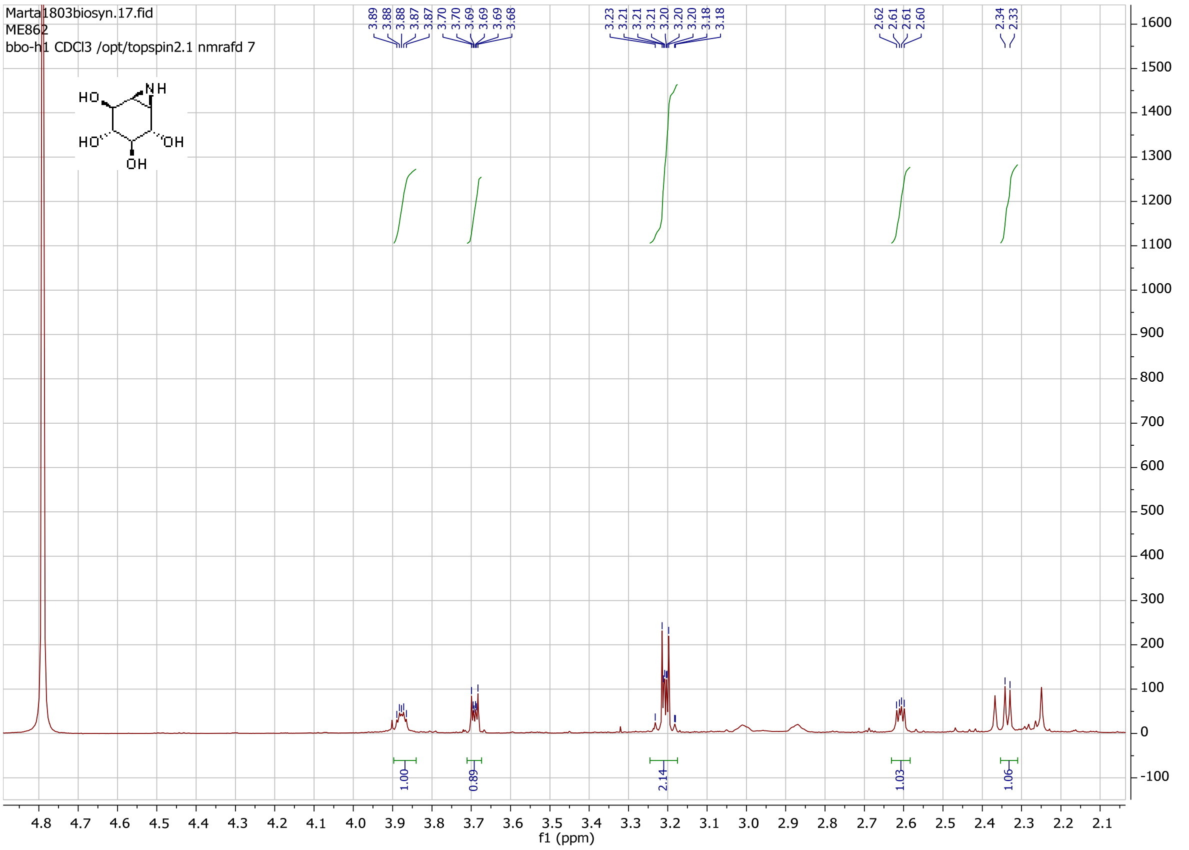

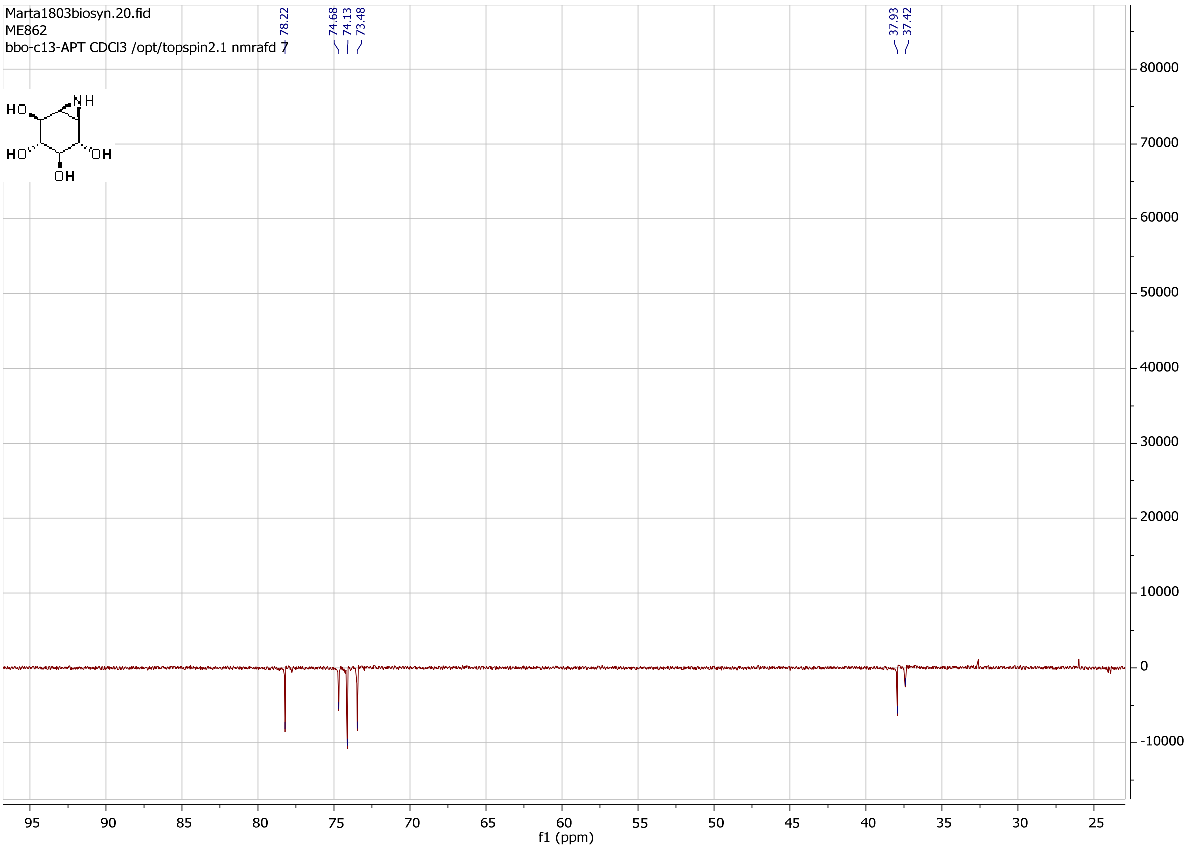

^{1}H-NMR and ^{13}C-NMR spectra of **8b** in $CDCl_3$

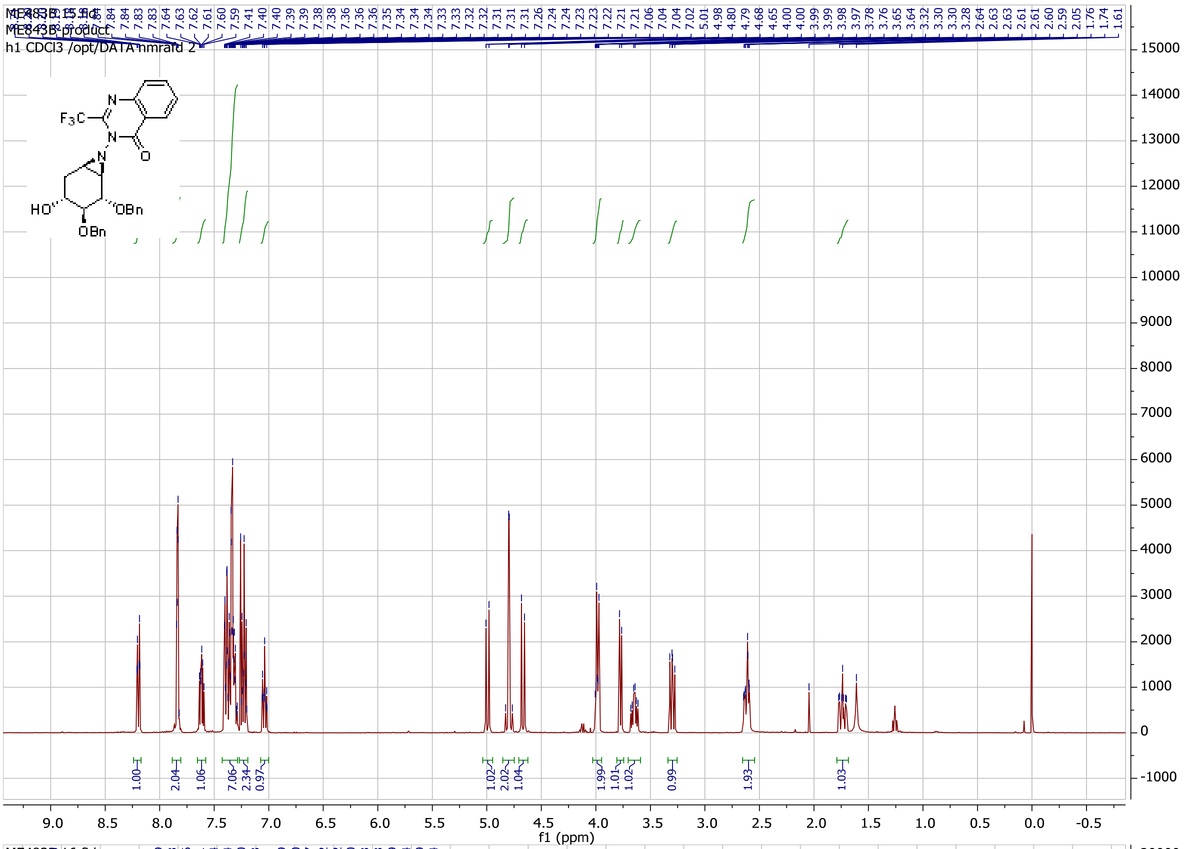

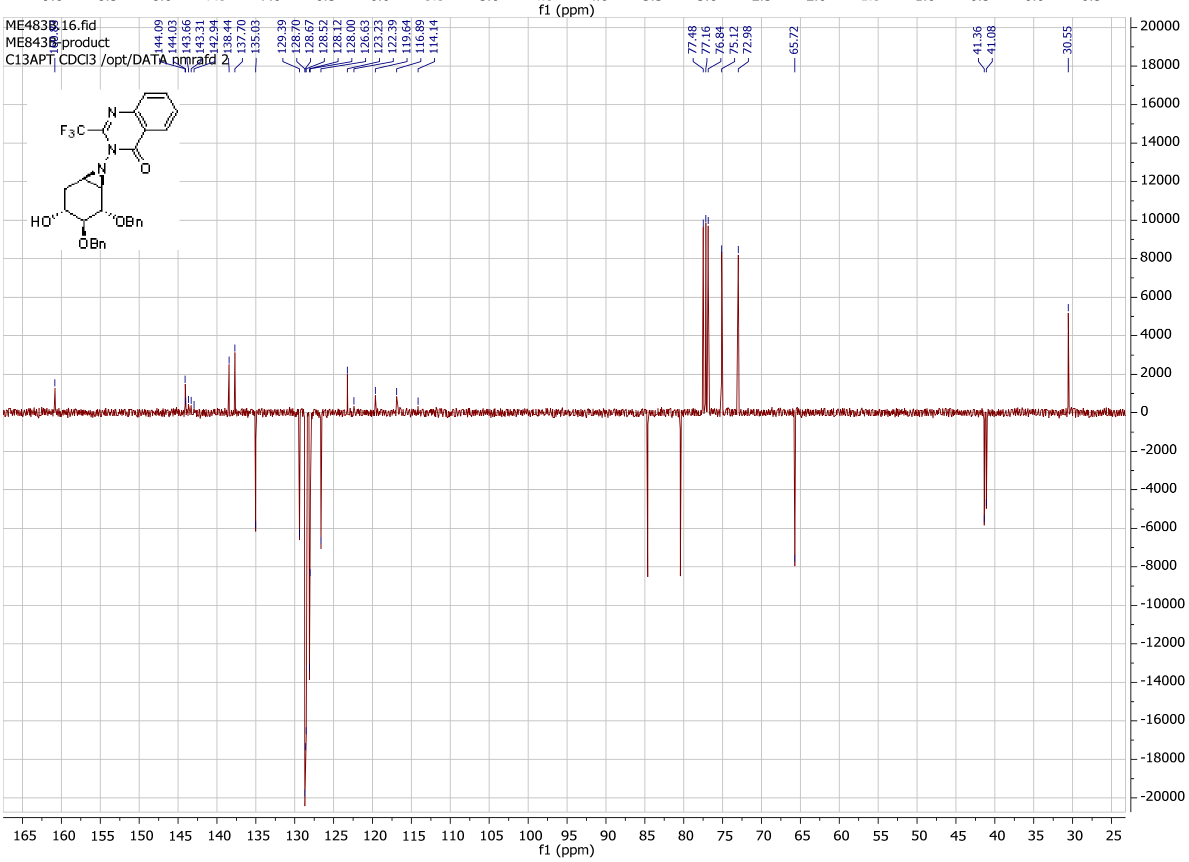

^{1}H-NMR and ^{13}C-NMR spectra of **8c** in D_2O

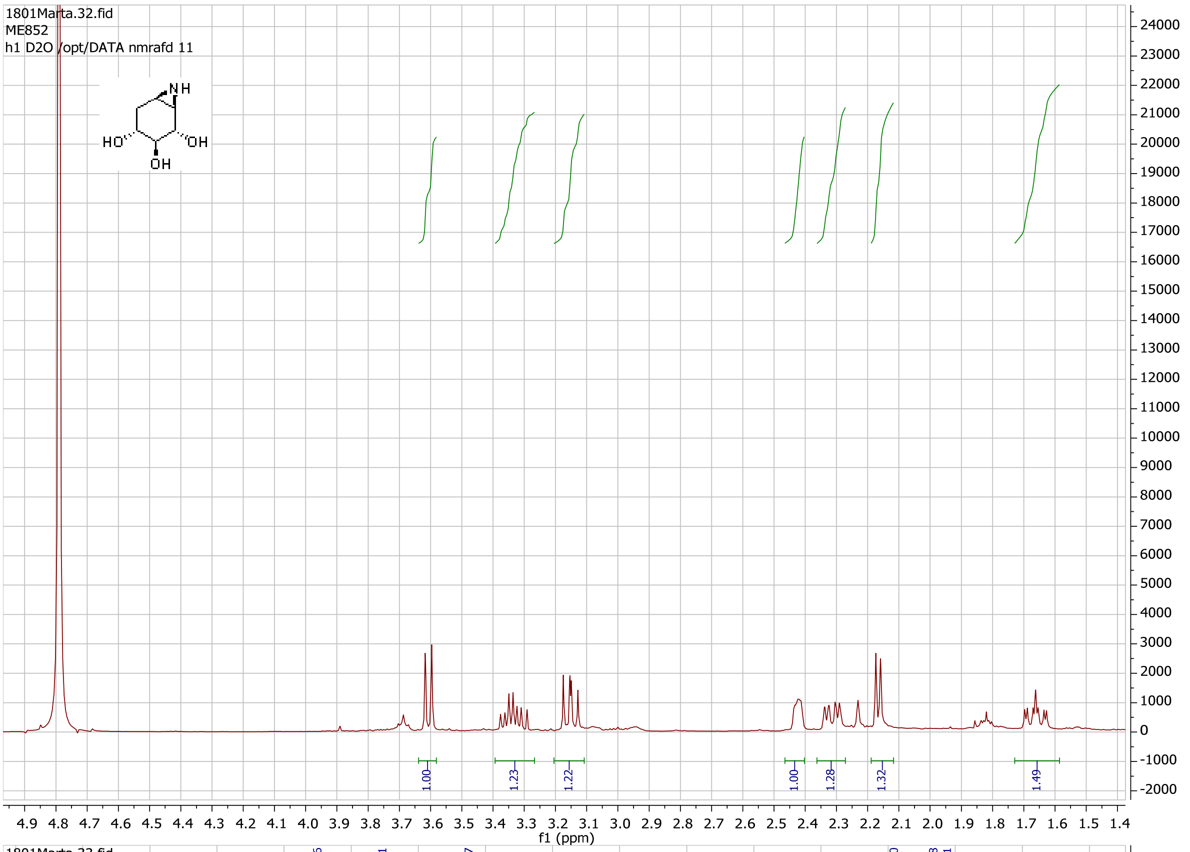

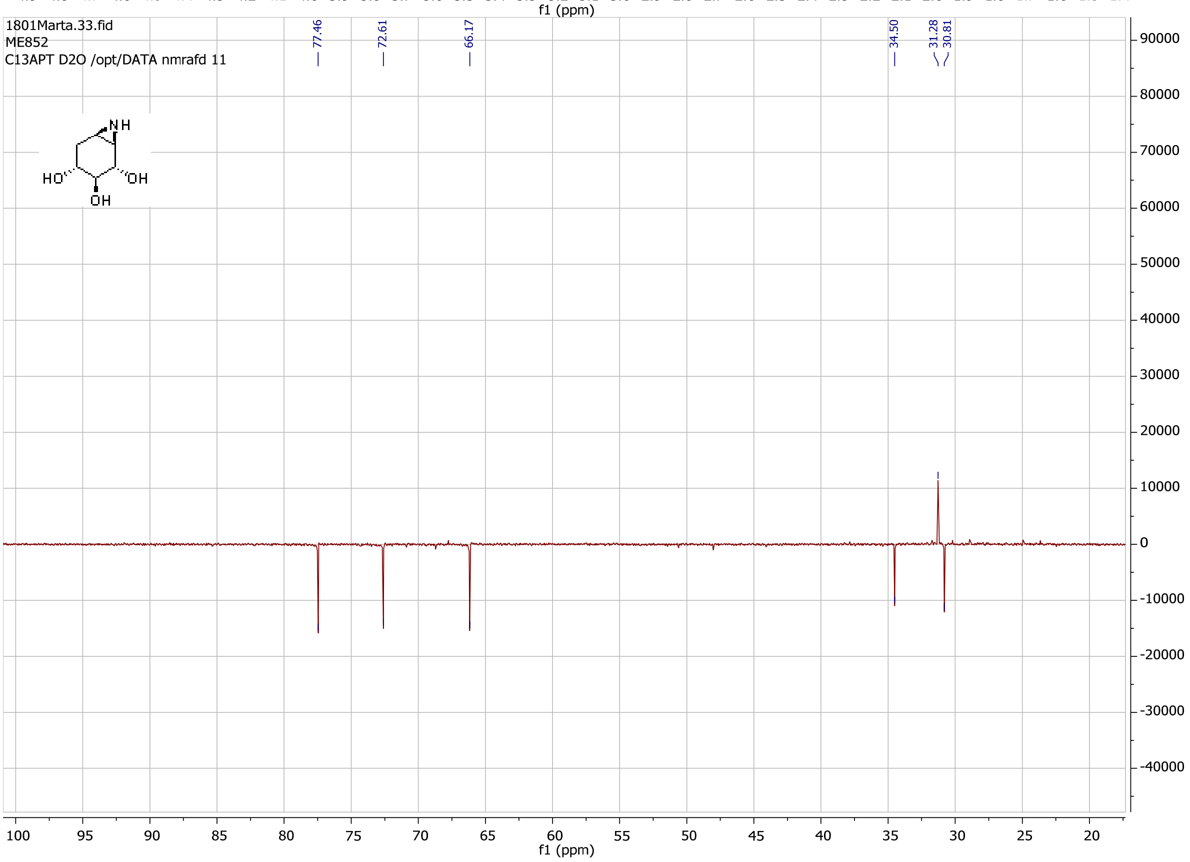

^{1}H-NMR and ^{13}C-NMR spectra of **9b** in $CDCl_3$

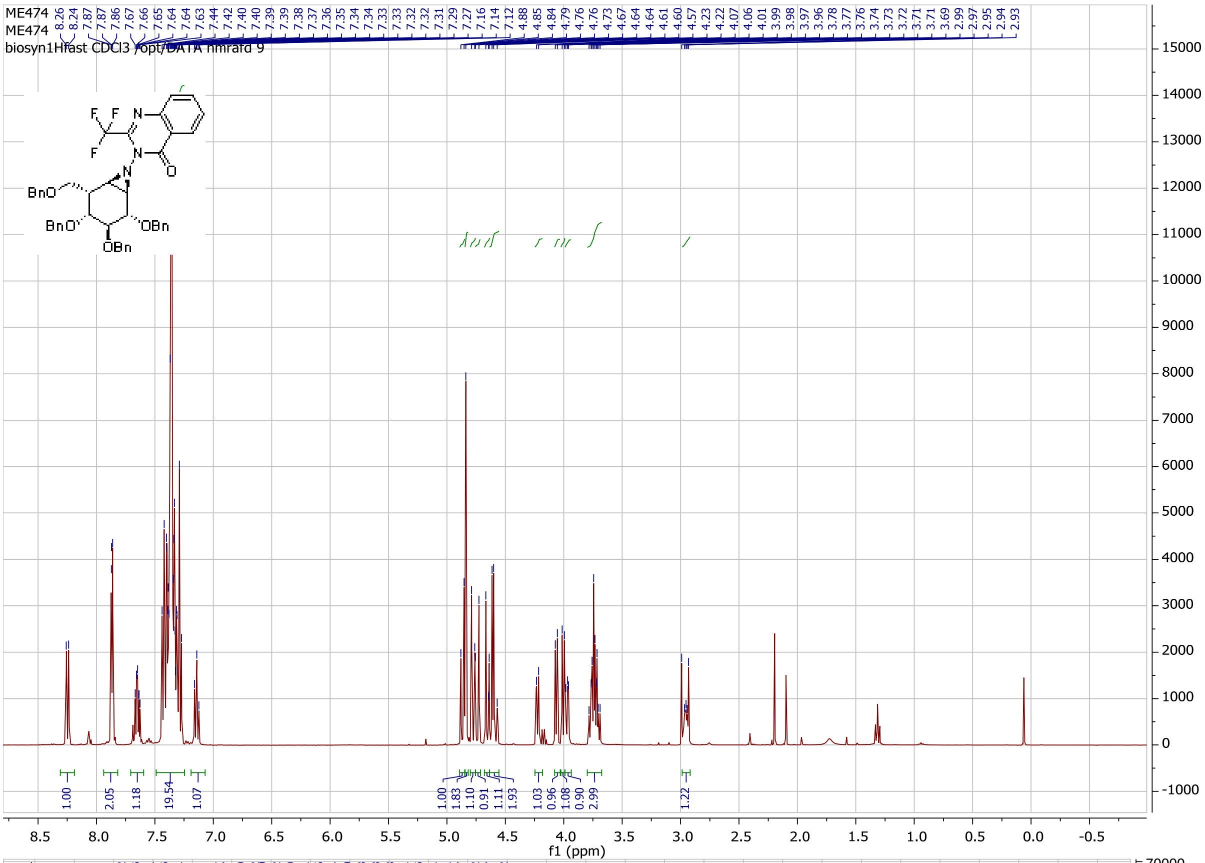

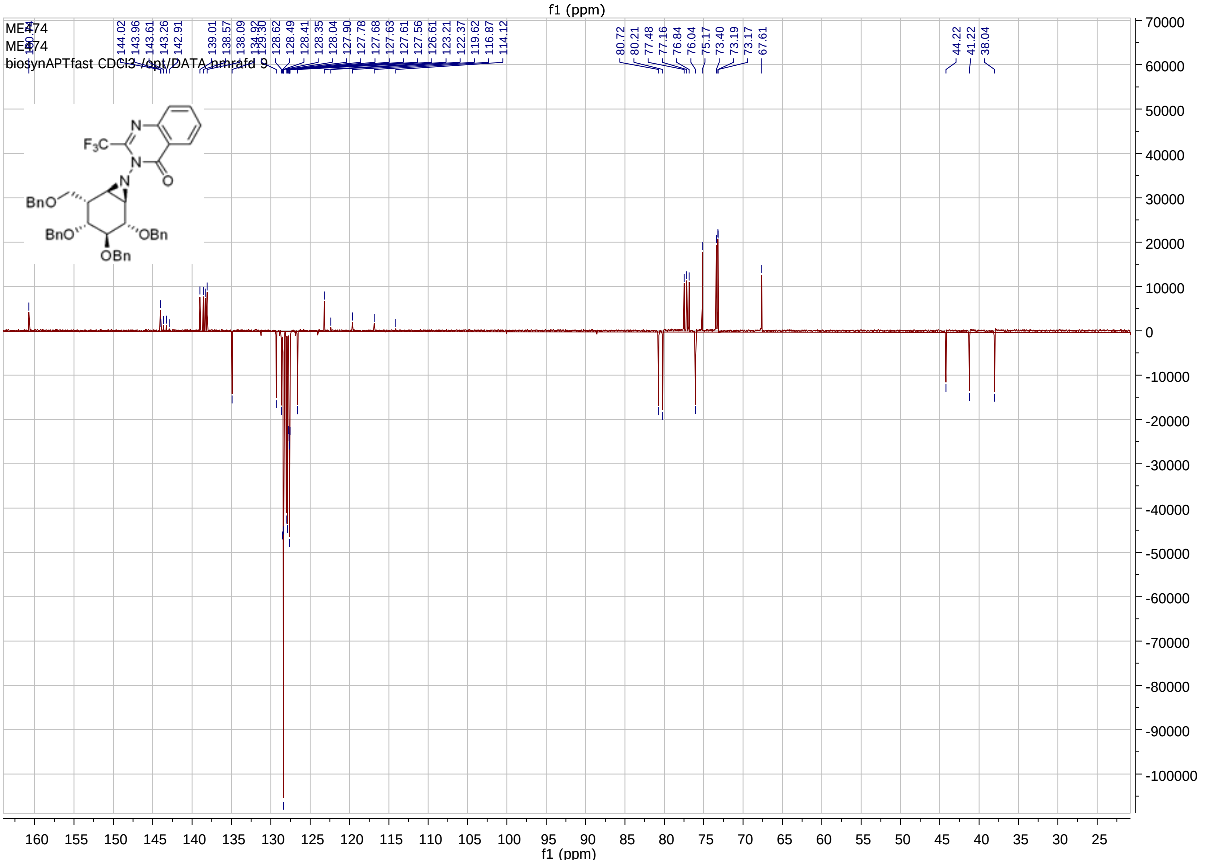

^{1}H-NMR and ^{13}C-NMR spectra of **10c** in D_2O

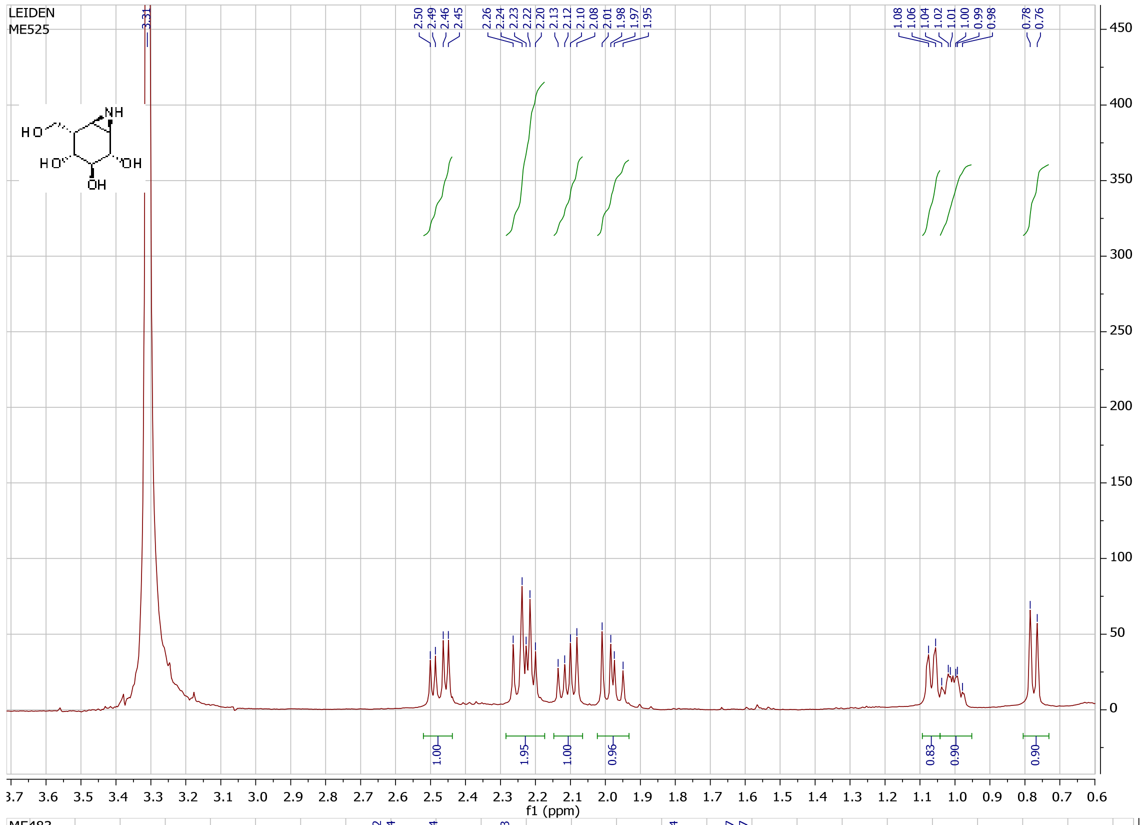

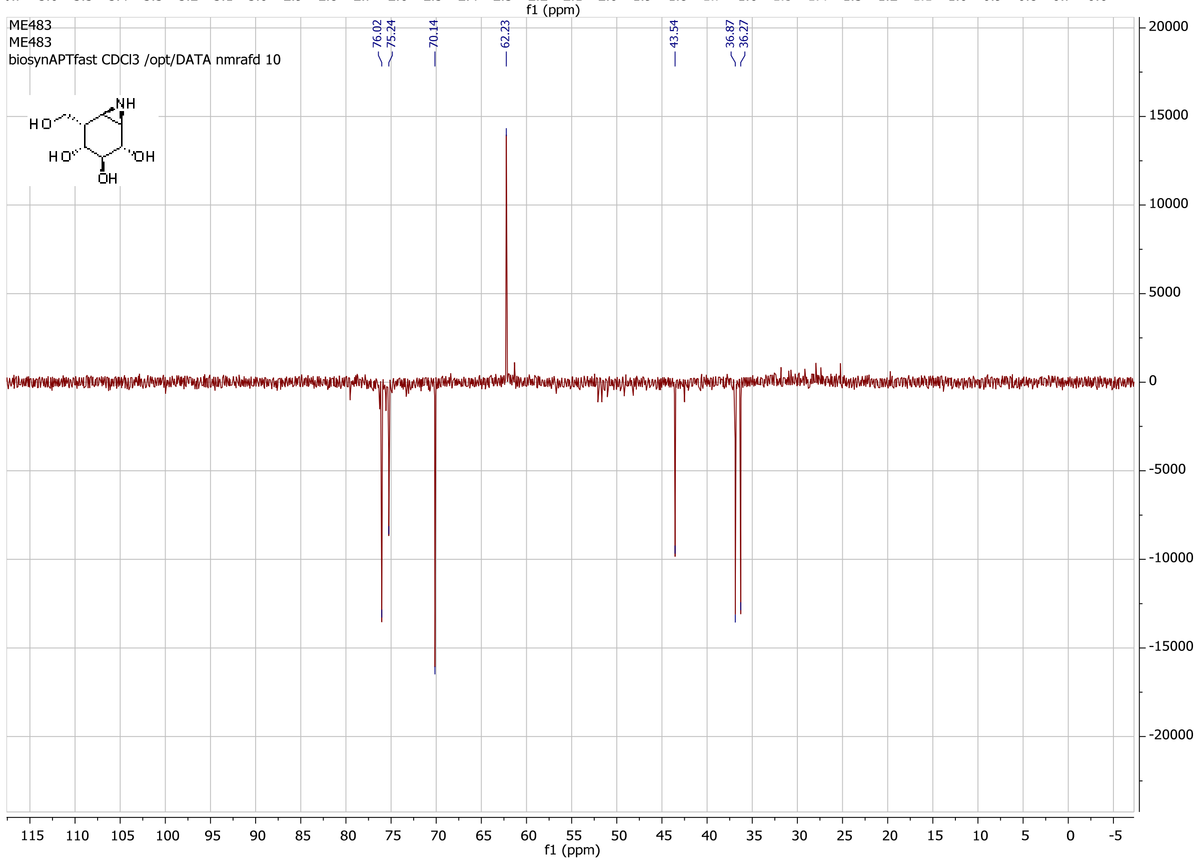

Supplement: Supplementary file 1 — Supporting Information [file EJOC-2019-1397-s001.pdf]
